# Extending the Scope of ^19^F Hyperpolarization through Signal Amplification by Reversible Exchange in MRI and NMR Spectroscopy

**DOI:** 10.1002/open.201700166

**Published:** 2017-12-21

**Authors:** Alexandra M. Olaru, Thomas B. R. Robertson, Jennifer S. Lewis, Alex Antony, Wissam Iali, Ryan E. Mewis, Simon B. Duckett

**Affiliations:** ^1^ Centre for Hyperpolarization in Magnetic Resonance, Department of Chemistry University of York Heslington YO10 5NY United Kingdom; ^2^ School of Science and the Environment, Division of Chemistry and Environmental Science Manchester Metropolitan University John Dalton Building, Chester St. Manchester M1 5GD United Kingdom

**Keywords:** ^19^F NMR spectroscopy, hyperpolarization, insensitive nuclei enhanced by polarization transfer (INEPT), magnetic resonance imaging (MRI), signal amplification by reversible exchange (SABRE)

## Abstract

Fluorinated ligands have a variety of uses in chemistry and industry, but it is their medical applications as ^18^F‐labelled positron emission tomography (PET) tracers where they are most visible. In this work, we illustrate the potential of using ^19^F‐containing ligands as future magnetic resonance imaging (MRI) contrast agents and as probes in magnetic resonance spectroscopy studies by significantly increasing their magnetic resonance detectability through the signal amplification by reversible exchange (SABRE) hyperpolarization method. We achieve ^19^F SABRE polarization in a wide range of molecules, including those essential to medication, and analyze how their steric bulk, the substrate loading, polarization transfer field, pH, and rate of ligand exchange impact the efficiency of SABRE. We conclude by presenting ^19^F MRI results in phantoms, which demonstrate that many of these agents show great promise as future ^19^F MRI contrast agents for diagnostic investigations.

## 1 Introduction

Hyperpolarized magnetic resonance spectroscopy (MRS) methods have been successfully used in conjunction with ^1^H, ^13^C, and ^15^ n detection.^1^ An important nucleus that could successfully be exploited in a similar manner is ^19^F. Its detection has already been shown to give remarkable results in traditional (i.e. thermal equilibrium) MRS applications.^2^ Furthermore, ^18^F radio‐labelled positron emission tomography (PET) agents are highly successful in the clinic as diagnostic probes.^3^

In the context of MRS applications, ^19^F presents several significant advantages over ^13^C and ^15^ n detection, as it is spin‐1/2 , 100 % abundant, and has 83 % of the sensitivity of ^1^H detection. Moreover, it has a very wide and, hence, diagnostic chemical shift range (much larger than those of ^13^C and ^31^P and comparable to that of ^15^ n) and it is not present endogenously in biological tissue, so there is no competing background signal to obscure the corresponding in vivo data. The large chemical shift range exhibited by hyperpolarized ^129^Xe nuclei reflects one of the reasons this alternative probe is attracting so much attention. So far, ^19^F MRS has been used to aid the probing of catabolic and anabolic drug conversions of anticancer agents such as 5‐fluorouracil^2^, ^4^ and the uptake and elimination of modern anesthetics used in preclinical magnetic resonance imaging (MRI).^5^ It has also been used for the noninvasive pharmacokinetic analysis of Voriconazole in the brain and plasma.^6^ Its large chemical shift range and high sensitivity to the local environment have been exploited in the assessment of cellular metal‐ion concentrations,^7^ tissue oxygenation,^8^ and pH.^9^

Various approaches to improve the typically low signal‐to‐noise ratio (SNR) of ^19^F MRI experiments are presented in the literature, such as increasing the number of ^19^F atoms per molecule. Variations in transverse relaxation times have also been exploited to provide molecular environment data.^10^ Hyperpolarization has been shown to be a very promising way of obtaining significantly stronger ^19^F signal compared to conventional methods. So far, the ^19^F nuclear magnetic resonance (NMR) response of various compounds has been successfully enhanced using both dynamic nuclear polarization^11^ and PHIP (*para*‐hydrogen‐induced polarization).^12^ The hyperpolarization of the ^19^F nucleus of 3‐fluoropyridine using signal amplification by reversible exchange (SABRE), which is also a *para*‐hydrogen (*p*‐H_2_)‐based technique, was reported in 2009 by Adams et al.^13^ More recently, Shchepin et al.^14^ presented the first hyperpolarized ^19^F SABRE MRI results using the same molecule and achieved signal enhancements of 100‐fold at 9.4 T by using SABRE‐SHEATH (shield enables alignment transfer to heteronuclei).^15^ Furthermore, a very recent report detailed the use of a superconducting quantum interference device (SQUID) for the simultaneous measurement of ^1^H and ^19^F spectra using SABRE.^16^ 3‐Fluoropyridine, ethyl‐5‐fluoronicotinic acid and 3,5‐*bis*(trifluoromethyl)pyridine were investigated. In addition, a further report described an attempt to utilize continuous hyperpolarization to hyperpolarize pentafluropyridine,^17^ but it was unsuccessful as the latter did not polarize. We seek to add to these early studies by screening a wide range of substrates to increase the applicability of this approach and suggest optimal routes to increasing ^19^F hyperpolarization by SABRE.

**Scheme 1:**
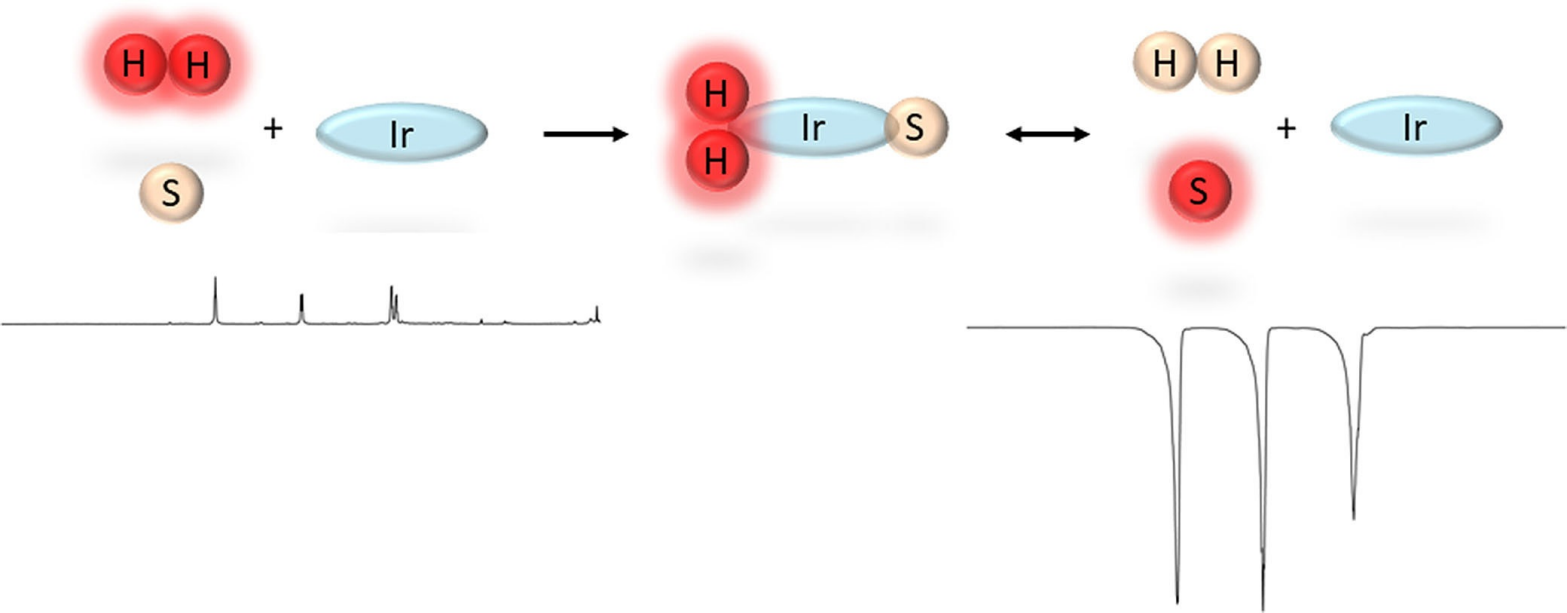
SABRE polarization‐transfer process: The substrate S and *p*‐H_2_ form temporary bonds with an iridium‐based catalyst. The polarization is transferred through scalar coupling from the *p*‐H_2_ to the substrate, which later dissociates. When interrogated through NMR methods, the free polarized substrate gives rise to enhanced signals.

SABRE utilizes *p*‐H_2_,^13^ which is a spin isomer of H_2_. As a nuclear singlet, it has no net spin and, so, it is unobservable from an NMR perspective. However, once *p*‐H_2_ has come into contact with a metal center, thereby forming *p*‐H_2_‐derived hydride ligands, this previously NMR invisible singlet state can be accessed. Now, polarization is transferred from the *p*‐H_2_‐derived nuclei to spin‐1/2 nuclei that are located in ligands attached to the same metal center through the *J*‐coupling network.^18^ The ligands that lie *trans* to the *p*‐H_2_‐derived hydrides receive polarization optimally, as they exhibit the largest difference in *cis* and *trans* couplings. The reversible nature of both H_2_ addition and analyte ligation at the metal center enables hyperpolarization to buildup in non‐ligated analyte molecules in a process that has been described as being catalytic in the transfer of polarization. The hyperpolarization that is created in this way can be readily read‐out in an NMR experiment and, hence, the signal intensity of the resulting NMR spectrum or magnetic resonance (MR) image is suitably enhanced (Scheme 1).

The transfer of polarization through SABRE typically occurs at low magnetic field and is maximized when a level anti‐crossing (LAC) condition is met.^19^ This is associated with the spin system becoming strongly coupled as a consequence of the chemical shift difference between the interacting groups collapsing to values that match the shared spin–spin couplings, thus resulting in coherent polarization transfer. Consequently, the efficiency of polarization transfer is field dependent. However, methods exist to transfer such polarization at high magnetic field in the presence of radio frequency excitation,^20^ such that hyperpolarized NMR spectra can be collected.^21^ Polarization transfer through SABRE has, in fact, been shown to be successfully transferred to ^1^H,^13^, ^22^ ^13^C,22a, 23 ^15^ n,^24^ ^19^F,^13^, ^14^, ^16^ ^31^P,^25^ ^29^Si, and ^119^Sn.^26^

**Scheme 2:**
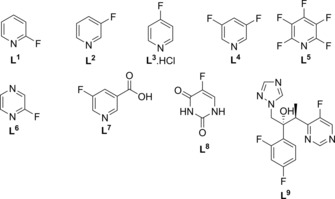
Ligands investigated in this work.

We further note that there are a large number of drugs that contain ^19^F nuclei; in 2010, it was calculated that about 20 % of administered drugs contained fluorine atoms or fluoroalkyl groups.^27^ In this work, we test a range of ^19^F‐containing substrates and drug molecules with a pyridyl arrangement (as depicted in Scheme 2) to demonstrate the possibility of applying SABRE in MR material and clinical investigations that rely on the response of ^19^F nuclei. Furthermore, we seek to determine the conditions required for maximizing the ^19^F NMR response of different ligands.

## 2 Results and Discussion

### 2.1 Formation of SABRE Polarization‐Transfer Catalysts

Prior to investigating the polarization transfer to the ^19^F nuclei of ligands **L^1^**–**L^6^** (Scheme 2) using the pre‐catalyst [Ir(IMes)‐ (COD)Cl]^28^ **1** (COD=cyclooctadiene; IMes=1,3‐bis(2,4,6‐trimethylphenyl)imidazole‐2‐ylidene), we probed the ability of **L^1^**–**L^6^** to displace the chloride ligand of **1** to give [Ir(IMes)(COD)L]^+^. This step has been shown to be important during SABRE activation and its absence would suggest a substrate that is unlikely to polarize.^29^ Unfortunately, attempts to isolate **L^3^**.HCl as the free base were unsuccessful under the conditions that we needed to employ the ligand. This is attributed to the fact that 4‐fluoropyridine reacts with itself to give *N*‐(4′‐pyridyl)‐4‐fluoropyridinium fluoride, which hydrolyzes to *N*‐(4′‐pyridyl)‐4‐pyridone (see Section 2.1 of the Supporting Information).^30^ Thus, this ligand was not investigated in detail. However, **L^1^**, **L^2^**, **L^4^**, **L^5^**, and **L^6^** were successfully employed.

A 256‐scan ^19^F NMR spectrum of 20 equivalents of **L^1^** or **L^5^** in the presence of **1** (5 mm) yielded only a single set of peaks, that of free **L^1^** or **L^5^**. A second set of peaks, owing to the formation of [Ir(IMes)(COD)L]^+^, was not observed. This contrasts strongly with the data obtained for **L^2^** under the same conditions, in which two peaks are observed in a ratio of 0.84:19.16 that correspond to [Ir(IMes)(COD)**L^2^**]^+^ and free **L^2^**, respectively. These two peaks are separated by 362 Hz in a 1.4 T NMR field. The same observation was made when **L^2^** was exchanged for **L^4^**; in this case, the two peaks were separated by 335 Hz in a 1.4 T NMR field, although the ratio was smaller (0.25:19.75 for **L^4^**/[Ir(IMes)(COD)**L^4^**]^+^). Conversely, when **L^5^** was used, the ^19^F NMR spectrum comprised of only three peaks, representative of the three different ^19^F NMR environments that **L^5^** possesses. Just like **L^1^**, there were no other observable peaks for bound **L^5^**. As both **L^1^** and **L^5^** possess *ortho*‐fluorine nuclei, there could be a possibility that steric hindrance around the nitrogen used to ligate to the metal center of **1** is preventing effective ligation. Compared to hydrogen, ^19^F has a greater van der Waals radius by 0.27 Å, but is still smaller than a methyl group (van der Waals radius 2.00 Å).^31^ Shchepin et al. reported that lutidines and picolines possessing a methyl group in the *ortho*‐position yielded no detectable ^15^ n hyperpolarization through SABRE‐SHEATH.^15^ In addition, the *p*K_a_ of **L^1^** (−0.44) is such that it may not be an effective ligand in comparison to pyridine (*p*K_a_=5.23) or 3‐fluoropyridine (*p*K_a_=2.97). To the best of our knowledge, no reported *p*K_a_ data are available for **L^4^**, although 3,5‐dichloropyridine has a reported *p*K_a_ of 0.51.^32^ **L^4^** would be expected to have a similar *p*K_a_ and, thus, is more basic than **L^1^** but less so than **L^2^**. Wang et al. indicated that the *p*K_a_ of **L^4^** was <2, as the hydrolysis rate of their ruthenium complexes increased relative to when pyridine or 3‐picoline were employed.^33^ **L^5^** is reported to not react with hot aqueous hydrogen iodide or hydrochloric acid, owing to the base‐weakening effect of the two *ortho*‐fluorines.^34^ The combination of steric as well as binding affinity to the metal center may preclude **L^1^** and **L^5^** acting as efficient ligands to **1. L^5^** has previously been reported as not being SABRE active.^17^

### 2.2 ^1^H SABRE Hyperpolarization of F‐Substituted Ligands

We then sought to evaluate the ability of **1** to form efficient SABRE polarization‐transfer catalysts with the ligands shown in Scheme 2. This is normally associated with the formation of [Ir(H)_2_(IMes)(**L**)_3_]Cl. **L^1^**, when examined under SABRE, produced a small enhancement (below unity) for both ligand loadings tested here (4 and 20 equivalents of **L** relative to **1**). Furthermore, a strong hydride signal for the corresponding complex [Ir(H)_2_(IMes)(**L^1^**)_3_]Cl was not observed. Attempts to increase the binding strength by using a smaller carbene (IMe) were unsuccessful (see Section 2.1 of the Supporting Information). Hence, **L^1^** is poorly suited to SABRE.

**Figure 1:**
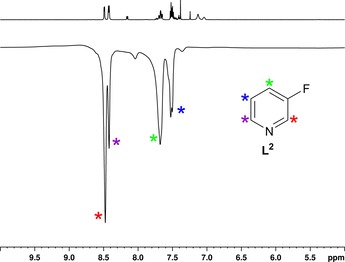
^1^H NMR spectra of **L^2^** (17‐fold excess) acquired in thermal equilibrium conditions (top, 128 scans) and hyperpolarized under SABRE (bottom, one scan).

In contrast, **L^2^** proved to provide a substantial ^1^H SABRE response. When the substrate loading is 1:4, ^1^H SABRE NMR spectra recorded after adding 3 bar of *p*‐H_2_ and shaking the sample for 10 s in a field of approximately 65 G show that the protons H‐2 and H‐6 of **L^2^** exhibit a 2103‐fold summed signal gain, which compares to an enhancement of −2397 reported for pyridine by Lloyd et al. under analogous conditions.^29^ When the metal/ligand ratio is 1:20, which corresponds to 17‐fold excess of ligand to catalyst (spectra presented in Figure 1), the corresponding values are 393‐fold for pyridine and 1296‐fold for **L^2^**, a value approximately six times higher than the one reported by Shchepin et al. for the same sample at 9.4 T.^14^ We attribute this significant difference to the difference in the purity of the *p*‐H_2_ gas used for sample polarization (96 % in this work and 50 % in Ref. 14). This suggests that **L^2^** is a good agent for SABRE in accordance with early observations^13^, ^14^ and confirms that the *p*‐H_2_ concentration has a significant effect on the efficiency of the polarization transfer process.

^1^H NMR spectra recorded after H_2_ addition and subsequent catalyst activation show that [Ir(IMes)(H_2_)(**L^2^**)_3_] is formed and is the dominant SABRE polarization‐transfer catalyst in solution, with a diagnostic hydride signal at *δ*−23.11 ppm. A second complex, indicated by a pair of hydride resonances *δ* at 23.87 and −25.15 ppm, was found to be [Ir(IMes)(H_2_)(**L^2^**)_2_(MeOH)] (see the Supporting Information for further details and characterization data). The possibility of Cl rather than methanol binding was excluded by acquiring spectra of the same reaction in dichloromethane and comparing the results. As exemplified elsewhere,^35^ methanol can be an active participant in the polarization‐transfer catalyst and, when employing mild acidic conditions, solvent polarization can be observed and quantified by measuring the enhancement of the O*H* resonance. Although evidence for methanol binding was obtained, no OH or residual CD_2_HOD signal enhancement was observed. Shchepin et al. presented ^1^H NMR spectra in their Supporting Information, which possess unattributed signals that may reflect the observation of species of this type.^14^

The activation parameters corresponding to the process of free ligand buildup in solution following dissociation from the dominant complex were calculated by using data obtained from a series of variable‐temperature exchange spectroscopy (EXSY) measurements (see Section 2.4 of the Supporting Information). The **L^2^** buildup rate at 300 K was extrapolated through Eyring analysis and was found to be 65.1 s^−1^, that is, approximately 2.8 times larger than the corresponding rate of pyridine measured using the same conditions.^29^ Furthermore, the enthalpy value for the buildup process of 98 kJ mol^−1^ suggests that the binding energy is slightly higher than that of pyridine (for which the site *trans* to hydride has a Δ*H* ^*≠*^_(build‐up)_ value of 95 kJ mol^−1^).

When considering the related molecules **L^4^**, **L^6^**, and **L^7^**, very good ^1^H SABRE polarization (of the order of hundreds up to thousands) was obtained in all cases. The SABRE polarization‐transfer catalysts formed with **1** in solution are the *tris*‐substituted species, [Ir(IMes)(H)_2_(L)_3_]^+^, and the analogous methanol complex, [Ir(IMes)(H)_2_(L)_2_(MeOH)]^+^ (see the Supporting Information for enhancement values, thermodynamic parameters, and characterization data).

A series of one‐shot ^1^H NMR spectra were collected on samples containing one‐fold and 17‐fold excesses of **L^7^** in the presence of 5 mm of **1** in MeOD solution. In the case of the sample containing a one‐fold excess, substantial signal enhancements were observed for all three of the non‐exchangeable resonances of the free substrate, with the largest ^1^H NMR signal enhancement being observed for H‐2 (−272±24), followed by H‐4 and H‐6 (−181±16 and −121±9 at 400 MHz, respectively). The corresponding values for the solution containing a 17‐fold excess of ligand were 59±2, 50±2, and 55±2. These values are much lower than those seen for **L^2^** and reflect the fact that the protonated form of the free ligand (**L^7^** **a**) dominates in solution.

Evidence for methanol binding to the iridium center was again seen in the hydride region of the associated ^1^H NMR spectra, which, in the case of **L^7^**, contains a resonance at *δ*−23.45 ppm, corresponding to the *tris*‐substituted complex. A pair of resonances, located at *δ*−23.84 and −24.04 ppm, arise from a complex where the equatorial sites are occupied by one substrate molecule, one methanol molecule, and two hydrides.

The corresponding ^1^H SABRE NMR experiments now result in a polarized O*H* resonance, exhibiting average enhancement values of 6.65±0.5 (one‐fold excess) and 51±2 (17‐fold excess). We have shown, in our previous work,35a that the deprotonation of the N center of the conjugate acid form of nicotinic acid and, subsequently, more efficient binding to **1**, can be achieved by adding a mild base to the solution. When analyzing samples containing 20 and 100 mm of **L^7^** after adding Cs_2_CO_3_ in equal amounts to the substrate (one‐fold and 17‐fold excess to **1**, respectively), the enhancement of the free‐ligand resonances increases considerably in both cases, as a result of the molecule being deprotonated (**L^7^** **b**) and increasing the ligand's probability of binding to the catalyst. We note, however, that in the case of low ligand loading, the presence of base promotes and accelerates H–D exchange, a phenomenon that occurs immediately after activation and on the timescale of the experiments, resulting in a progressive decrease of the total enhancement with each addition of fresh *p*‐H_2_. Data shows that at least 40 % deuteration of the three sites takes place in the first 15 minutes (see Section 2.2 of the Supporting Information).

**Figure 2:**
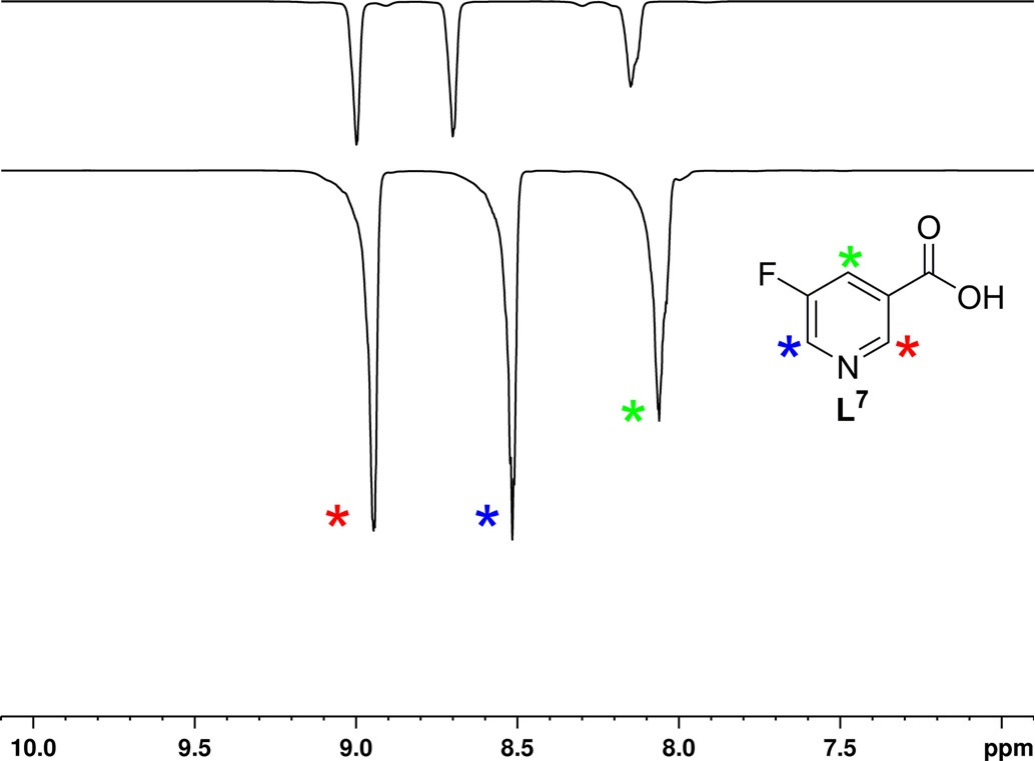
^1^H NMR single‐shot SABRE hyperpolarized spectra of 5‐fluoronicotinic acid before (**L^7^** **a**, top) and after (**L^7^** **b**, bottom) addition of Cs_2_CO_3_ for a sample prepared by using a 17‐fold excess of ligand to catalyst.

For the sample prepared using a one‐fold excess of ligand and a one‐fold excess of base, relative to the catalyst, the total signal enhancement obtained across the three sites was 2.5‐times higher than that seen for a similar sample prepared without base; the corresponding difference increased to 4.5 times for the 17‐fold loading (spectra presented in Figure 2). Furthermore, neither methanol binding nor OH signal enhancement was observed when the base was present. These results relate to those reported for niacin35a and confirm the importance that the pH can play in manipulating substrate binding.

**Figure 3:**
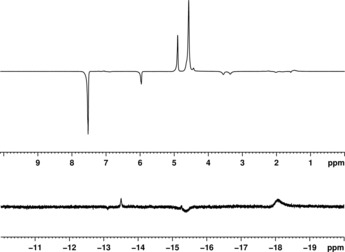
^1^H NMR single‐shot SABRE hyperpolarized spectrum of **L^8^**: aromatic region (top) and hydride region (bottom).

We have also analyzed the fluorine‐substituted nucleobase used widely used in cancer therapy, 5‐fluorouracil (**L^8^**, commonly sold under the commercial name of Adrucil), and the *anti*‐fungal drug Vorincazole (**L^9^**), both of which are listed on the World Health Organisation's essential medicines list. As **L^8^** is present in solution in its fully protonated form (thus preventing the N centers from binding to **1**), we adopted a strategy similar to the one used for **L^7^** to promote the formation of the SABRE polarization‐transfer catalyst and to improve the signal enhancements. In the hydride region of this ^1^H NMR spectrum, we initially see two signals at *δ*−15.4 and −18.1 ppm, which are in emission and absorption, respectively, owing to the formation under ALTADENA (adiabatic longitudinal transport after dissociation engenders nuclear alignment)^36^ conditions (Figure 3). We note that these observations are consistent with other reports that have considered the activation of SABRE catalysts.^37^ In addition, we see two SABRE enhanced C*H* resonances belonging to a bound COE (cyclooctene) ligand at *δ* 3.57 and 3.35 ppm, which is consistent with a prior report,37a as well as a weakly polarized peak corresponding to the mesityl protons of the carbene.

Hence, the initial product, [Ir(H)_2_(IMes)(COE)(**L^8^**)_2_]Cl, exhibits significant SABRE activity. The signal enhancement reduces over the next 5 minutes, as this complex is converted into multiple species (see the Supporting Information for detailed information). Under these conditions, when the excess of **L^8^** is fourfold and 0.5‐fold excess of Cs_2_CO_3_ is present, we see a signal at *δ* 7.56 ppm, corresponding to the free resonance of H‐5. This signal possesses an initial enhancement of 53‐fold and a second peak at *δ* 5.97 ppm, corresponding to the H‐5 resonance of the ligand when bound to the Ir center, shows an initial 26‐fold enhancement. A set of optimization experiments were undertaken to identify the best base and ligand concentrations, and ultimately a 100‐fold ^1^H NMR signal enhancement was obtained for the free H‐5 resonance of **L^8^** and approximately a 57‐fold enhancement was observed for the peak corresponding to the bound ligand. Unfortunately, these values decrease rapidly with time, owing to a process of deactivation that quenches the activity of the catalyst. Attempts to block this cyclometalation process using a smaller carbene were unsuccessful under the conditions employed. We also note that rapid H/D exchange leads to the formation of CD_3_OH alongside the deuteration of the iridium hydride.

Polarization transfer of four equivalents of **L^9^** using **1** in the presence of *p*‐H_2_ in earth's magnetic field was also probed. The pyrimidine ring protons were enhanced twofold. Compared with the other ligands studied, **L^9^** possesses the greatest steric bulk, and so this could be the main factor for the low enhancement obtained. When the ratio of **L^9^**/**1** was increased to 20:1, SABRE enhancement of the pyrimidine ring protons was not detectable.

Hence, we can conclude that very high levels of ^1^H hyperpolarization can be achieved in ^19^F‐containing materials under SABRE, with the exception of **L^9^**.

### 2.3 ^19^F SABRE Hyperpolarization of F‐Substituted Ligands

**Figure 4:**
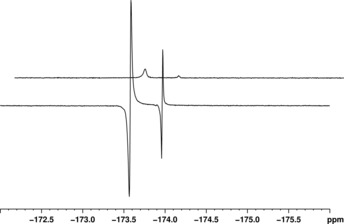
^19^F NMR single‐shot spectra of **L^8^** (7‐fold excess) acquired in thermal equilibrium conditions (top) and hyperpolarized under SABRE (bottom).

We have investigated the ^19^F hyperpolarization of the ligands analyzed in this work by using a 500 MHz spectrometer (11.74 T) equipped with dual capacity ^1^H–^19^F high‐resolution probe and acquired ^19^F hyperpolarized spectra using π/2 pulses immediately after shaking the samples in the stray field of the magnet. As was the case for ^1^H, **L^2^** provided the best response, with enhancements of approximately 60 for the free resonance (*δ* −127.5 ppm) and 36 for the bound equatorial signal (*δ* −124.1 ppm). We note that the signal is *anti*‐phase when polarization is conducted in the fringe field of the magnet, as a longitudinal two‐spin order ^1^H–^19^F term is created, which is analogous to the homonuclear ^1^H–^1^H term that is created under in‐field PHIP,^38^ and so enhancements are calculated by using magnitude mode. Lower values were obtained for the other substrates considered (see Section 4 of the Supporting Information for ^19^F NMR spectra and full enhancement data). Interestingly, the ^19^F signal of **L^4^** is entirely in‐phase, whereas, when SABRE‐SHEATH is employed, the signal becomes *anti*‐phase in character. Conversely, the opposite is true for **L^2^**. An average enhancement for **L^8^** could not be calculated, owing to the fast evolution of the cyclometalation process, but both the free and the bound resonance are enhanced and display an antiphase behavior (Figure 4).

**L^9^** resulted in a fivefold enhancement for the ^19^F nucleus of the pyrimidine ring when a ratio of 4:1 **L^9^** to **1** was employed. This was observed as an *anti*‐phase signal. However, no polarization was observed for the other two ^19^F nuclei, owing to insufficient *J* coupling to propagate polarization to these two nuclei. SABRE‐SHEATH methods did not yield any substantial increase in the polarization observed, although they did convert the signal from *anti*‐phase to in phase (see the Supporting Information). Despite the low enhancement observed, the *T*_1_ of the polarized ^19^F resonance is 5.15 s in a measurement field of 1.4 T. This is comparable to the *T*_1_ observed for a ratio of 20:1 **L^9^**/**1** at 11.74 T for the other substrates studied (see Table 2).

Although the results of the ^19^F hyperpolarization studies are very promising and exhibit enhancement values high enough to allow for high‐resolution images (with the exception of **L^9^**) to be recorded (see below), they are two orders of magnitude lower than those obtained for ^1^H. This is a remarkable decrease considering the high sensitivity of ^19^F, which is very close to that of proton. In order to fully rationalize this difference, one must take into account the relaxation and exchange rates for the ligands analyzed, as well as the dependence of the polarization transfer on the value of the magnetic field at which the transfer takes place.

### 2.4 ^1^H and ^19^F Relaxation Times

We have measured and compared the ^1^H and ^19^F *T*_1_ values for each ligand, as it is widely known that relaxation is one of the main factors affecting the efficiency of the SABRE hyperpolarization transfer process. When examining the ^1^H longitudinal relaxation times of the ligands prepared in MeOD solution in the presence of the polarization‐transfer catalyst (17‐fold excess of substrate to Ir), we have found that the *T*_1_ values range between 15 and 45 s, most of them being above 20 s (measured at 9.4 T). The data, presented in Table 1, show that all substrates analyzed exhibit relatively long relaxation times, higher or comparable to those of pyridine. The lowest values measured correspond to **L^7^** in its protonated form, probably owing to the presence of a hydrogen atom on the binding center.

**Table 1:** ^1^H longitudinal relaxation times (*T*_1_ [s]) of the individual protons of the substrates tested, measured at 9.4 T on samples prepared using 5 mm of **1** and 17‐fold excess of ligand.

Proton	**L^2^**	**L^4^**	**L^6^**	**L^7^** **a**	**L^7^** **b**	Pyridine^[a]^
H‐2	19.03	21.90	–	14.52	46.14	12.6
H‐3	–	–	22.59	‐	–	14.7
H‐4	13.47	29.24	–	14.54	16.04	18.9
H‐5	21.11	–	25.28	–	–	14.7
H‐6	11.56	21.90	27.09	12.73	16.91	12.6

[a] As reported by Lloyd et al.^29^

As shown in our previous work,35a addition of base and the subsequent change in pH has a favorable effect on the *T*_1_ of H‐2 of **L^7^**, which, in this case, increases remarkably from 14.5 to 46.1 s. Smaller increases can also be noted for protons H‐4 and H‐6.

When performing similar experiments to determine the corresponding ^19^F *T*_1_ values, we have found that they lie between 3.3 s (**L^7^** **b**) and 5.5 s (**L^4^**) when measured at 11.74 T under Boltzmann equilibrium conditions. This significant difference in relaxation rates between proton and fluorine can at least partially account for the much lower enhancements obtained for ^19^F in comparison to ^1^H. We also note that these values are smaller than those reported by Shchepin et al. for hyperpolarized 3‐fluoropyridine at 9.4 T. However, a second set of *T*_1_ data collected at 1.4 T for **L^4^** and **L^2^** reports that the *T*_1_ values of the ^19^F nuclei are far longer than at 11.74 T by a factor of 3–5 times. This is attributed to the relaxation process being dominated by chemical shift anisotropy rather than dipolar relaxation and can, therefore, be expected to scale with *B*_0_. In the context of SABRE‐based experiments, a similar effect has previously been observed for ^15^ n,^39^ and more recently ^19^F.^14^ Consequently, these data motivated the collection of ^19^F hyperpolarized NMR spectra of **L^2^** (4 equiv relative to **1**) at 1.4 T. Relative to a thermal trace, this spectrum revealed that the enhancement for **L^2^** was now 244‐fold (calculated using magnitude data), an improvement of four times over those collected at 11.74 T. Furthermore, employment of SABRE‐SHEATH converted the *anti*‐phase signal into a 97 % in‐phase signal, but reduced the signal enhancement to 93‐fold. From an imaging perspective, the appearance of the signal is important because broadened lines can lead to partial signal cancellation.

In a further investigation, we also probed **L^4^** in a similar fashion, but also utilized our knowledge of the kinetic exchange rates to inform our experimental protocol. Thus, the solution was cooled to 0 °C prior to hyperpolarization in a μ‐magnetic shield or at earth's magnetic field. No change was evident for the measurements that were polarized at earth's magnetic field, whereas employing SABRE‐SHEATH at 0 °C yielded a nine‐fold improvement over those at room temperature. Furthermore, the ^19^F signal is now completely in‐phase, whereas the analogous spectrum collected following polarization transfer at earth's magnetic field displays *anti*‐phase character.

The relatively long ^1^H longitudinal relaxation times (Table 2) were exploited in INEPT experiments, in which the ^1^H polarization was transferred to ^13^C and to ^19^F nuclei, respectively. In the case of ^13^C nuclei, this led to a significant increase in the enhancement values compared to the results obtained in Boltzmann equilibrium conditions, allowing us to record high‐quality ^13^C spectra in less than one second (see Section 3 of the Supporting Information). We also note that, in the case of **L^2^** and **L^7^** **b**, the use of an INEPT sequence for the acquisition of hyperpolarized ^19^F spectra led to a signal gain of approximately 230‐ and 100‐fold, respectively, when the results obtained using *p*‐H_2_ are compared with the INEPT spectra acquired in Boltzmann equilibrium conditions (see Section 4 of the Supporting Information for spectra and full data).

**Table 2:** ^19^F longitudinal relaxation times (*T*_1_ [s]) of the fluorine resonances of the substrates tested, measured on samples prepared using 5 mm of **1** and 17‐fold excess of ligand.

	**L^2^**	**L^4^**	**L^6^**	**L^7^** **a**	**L^7^** **b**
11.74 T	4.46	5.51	4.91	3.16	3.28
1.4 T	23.56	18.21	–	–	–

### 2.5 ^19^F MRI Results

To assess the potential of the ligands studied in this work as MR contrast agents for medical investigations, we have undertaken a series of MRI experiments performed on phantoms containing 5 mm of **1** and 100 mm of substrate dissolved in MeOD. Following *p*‐H_2_ addition, the samples were shaken in the stray field of the magnet and, after being in the imaging probe head, were quickly (after ca. 10 s) inserted into the spectrometer. One‐shot ^19^F MR images were acquired by using a double resonance ^13^C–^19^F 30 mm birdcage coil (^19^F frequency 376.5 MHz), employing a RARE acquisition protocol (see Section 5 of the Supporting Information for full experimental data).

SNR values have been calculated for each image by dividing the mean signal value by the standard deviation of the noise. The increase in SNR obtained by using SABRE was evaluated by comparing the values obtained for the hyperpolarized image with the SNR obtained for images acquired in Boltzmann equilibrium conditions (see Section 5 of the Supporting Information). The best results were obtained when using **L^2^**, for which the hyperpolarized results exhibited a 104‐fold maximum increase in SNR and an average gain of 100‐fold when compared with the image acquired in Boltzmann equilibrium conditions (see Section 5 of the Supporting Information for details).

**Figure 5:**
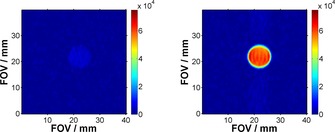
^19^F MRI SABRE hyperpolarized images of **L^7^** **a** (left) and **L^7^** **b** (right). For comparison purposes, the same range has been used for the intensity image data.

Furthermore, the MRI experiments emphasize the effect of deprotonation on the polarization‐transfer efficiency: while the hyperpolarized images of **L^7^** **a** have a very low intensity and exhibit no increase in SNR compared to the thermal counterpart, the values obtained for **L^7^** **b** show that SABRE hyperpolarization lead to a 75‐fold SNR gain compared to the result acquired in Boltzmann equilibrium conditions (Figure 5).

We believe that these results would be significantly improved if the time taken to transfer the sample into to the magnet for observation could be reduced (i.e. the amount of time between polarization and detection), as a significant amount of signal is lost during the 10 s interval (ca. 2×*T*_1_) imposed here by our hardware limitations.

## 3 Conclusions

We have evaluated the capacity of a wide range of fluorinated molecules to react with [IrCl(IMes)(COD)] (**1**) and H_2_ to successfully form a SABRE‐active catalyst that facilitates polarization transfer to ^19^F. This builds on the earlier reports of Adams et al. and Shchepin et al.^13^, ^14^ We have found that 2‐fluoropyridine (**L^1^**) and pentafluoropyridine (**L^5^**) react weakly, if at all, with the catalyst precursor. As both molecules possess *ortho*‐fluorine nuclei, there could be a possibility that steric hindrance around the nitrogen used to ligate to the metal center of **1** is preventing effective ligation. In the case of 4‐fluoropyridine.HCl (**L^3^**.HCl), attempts to isolate the base were unsuccessful and this ligand was not investigated in detail. SABRE polarization‐transfer catalysts were formed by using **L^2^**, **L^4^**, **L^6^**, **L^7^**, **L^7^**, and **L^9^** through reaction with **1** in the presence of H_2_; in the case of **L^7^** and **L^8^**, the formation of the activated catalyst was promoted by deprotonating the N center of the conjugate acid using a mild base (Cs_2_CO_3_). The performance of the new SABRE catalysts was analyzed as a function of ligand loading, polarization transfer field, temperature (**L^4^**), and pH (**L^7^**).

When comparing the ^1^H‐hyperpolarized NMR results obtained on the fluorinated compounds studied in this work with the ones obtained using pyridine, we find that when working at low ligand loadings (one‐fold excess of substrate), the total enhancement obtained for each molecule is considerably lower than for pyridine. However, when moving to high ligand loadings (17‐fold excess of substrate), the performance of pyridine is surpassed by **L^2^** and **L^6^** (with enhancements which are 3.2 and 1.2 higher), whereas the enhancements obtained for **L^4^** and **L^7^** **b** become comparable to the ones of pyridine. We show that this can be explained by considering the effect of relaxation in these fluorinated ligands, which relax slower, on average, than pyridine under the same conditions. We note that, in the case of **L^7^**, deprotonation using Cs_2_CO_3_ leads to a remarkable increase in *T*_1_ of the proton located between the N center and the carboxylic group, from 14.5 to 46.1 s. Our work demonstrates that, by varying the ligand loading and *p*‐H_2_ concentration, it is possible to dramatically exceed the previously reported ^1^H hyperpolarization levels. ^19^F NMR hyperpolarized spectra were successfully recorded for **L^2^**, **L^4^**, **L^6^**, **L^7^** **a**, **L^7^** **b**, **L^8^**, and **L^9^**, and the average enhancements of the free fluorine resonance, with the exception of **L^8^** and **L^9^**, were quantified. The highest values were obtained for **L^2^** and **L^7^** **b** (ca. 60‐ and 39‐fold enhancements, respectively, at 11.74 T), although we note that the enhancement values reported by Shchepin et al.^14^ exceed the ones reported here. However, we show that, by decreasing the field strength at which experiments are performed and increasing the concentration of *p*‐H_2_, the enhancement of the ^19^F resonance can be significantly improved.

The two orders of magnitude difference between the enhancements obtained for ^1^H and ^19^F can be explained by taking into account the short longitudinal relaxation times of ^19^F, which range between 3 and 5 s at 11.74 T for all of the ligands investigated here. Although this is apparently an obstacle in the way of ^19^F SABRE MRI applications, we show that, in some cases, one can circumvent this situation by exploiting the long relaxation times of the protons in INEPT polarization‐transfer experiments or by working at relatively low detection fields.

Furthermore, hyperpolarized 2D ^19^F MRI images have been recorded by using a similar polarization‐transfer procedure to that used in NMR spectroscopy experiments. When comparing the results with their Boltzmann equilibrium counterparts, we find that hyperpolarization facilitates an SNR gain of over one order of magnitude for most of the substrates investigated, particularly **L^2^** and **L^7^** **b**, for which the maximum SNR gains obtained were approximately 104 and 76, respectively, representing a very promising for future diagnostic MRI applications.

This work demonstrates the feasibility of using SABRE to hyperpolarize a wide range of ^19^F‐substituted ligands, including drugs included on the WHO list of essential medicines. We present various methods, through which the polarization of ^19^F nuclei can be optimized and we show, using NMR and MRI results, that some of the substrates investigated in this work exhibit tremendous potential for future applications such as clinical MRI investigations, molecular imaging, and in vivo pH assessment.

## Experimental Section

^1^H and ^13^C NMR measurements were recorded on a Bruker Avance III series 400 MHz spectrometer, whereas ^19^F NMR measurements as well as *T*_1_ inversion recovery and characterization experiments were performed on a Bruker Avance III series 500 MHz system. Samples concerning **L^9^** and the *T*_1_ measurements at 1.4 T were collected by using an Oxford Instruments Pulsar equipped with a ^1^H and ^19^F probe. NMR samples were prepared containing 5 mm catalyst precursor in 0.6 mL of [D_4_]MeOH. NMR measurements were collected using either 4 or 20 equivalents of substrate to 5 mm of iridium in 0.6 mL MeOD (leading to samples containing one‐ and 17‐fold excesses of ligand relative to iridium, respectively). *p*‐H_2_ was prepared by cooling hydrogen gas over charcoal at 20 K for all samples, except those of **L^9^** and measurements made at 1.4 T; these utilized hydrogen gas over charcoal at 77 K. After adding *p*‐H_2_ at 3 bar pressure, ^1^H NMR spectra were recorded by using π/2 excitation pulses immediately after shaking the sample in a magnetic field of 65 G. A similar procedure was used for the polarization of heteronuclei. For more details about the sample preparation and experimental procedures, please see Section 1 of the Supporting Information.

## Conflict of interest

*The authors declare no conflict of interest*.

## Supporting information

As a service to our authors and readers, this journal provides supporting information supplied by the authors. Such materials are peer reviewed and may be re‐organized for online delivery, but are not copy‐edited or typeset. Technical support issues arising from supporting information (other than missing files) should be addressed to the authors.

Supplementary
