# Supplementary material for: Extending the Scope of 19F Hyperpolarization through Signal Amplification by Reversible Exchange in MRI and NMR Spectroscopy
Source: ChemistryOpen. 2017 Dec 21;7(1):97–105. doi: 10.1002/open.201700166 (PMC5754555; doi:10.1002/open.201700166)
Supplement: Supplementary file 1 — Supplementary [file OPEN-7-97-s001.pdf]

ChemistryOPEN

Supporting Information

© 2018 The Authors. Published by Wiley-VCH Verlag GmbH & Co. KGaA, Weinheim

Extending the Scope of ^{19}F Hyperpolarization through Signal Amplification by Reversible Exchange in MRI and NMR Spectroscopy

Alexandra M. Olaru,[a] Thomas B. R. Robertson,[b] Jennifer S. Lewis,[a] Alex Antony,[b] Wissam Iali,[a] Ryan E. Mewis,*[b] and Simon B. Duckett*[a]

open_201700166_sm_miscellaneous_information.pdf

Supporting Information

1. Materials and Methods

2. ^{1}H NMR Experimental Results

    2.1 ^{1}H NMR SABRE polarization of model N-heterocyclic fluorinated compounds.

    2.2 ^{1}H NMR SABRE polarization of biologically relevant fluorinated compounds by pH manipulation.

    2.3 ^{1}H longitudinal relaxation times.

    2.4 Exchange rates measured as a function of temperature.

    2.5 ^{1}H NMR SABRE enhancements as a function of the polarization transfer field.

3. ^{13}C NMR Experimental Results

4. ^{19}F NMR Experimental Results

    4.1 ^{19}F NMR SABRE polarization of N-heterocyclic fluorinated compounds measured at 11.7 T.

    4.2 ^{19}F NMR SABRE polarization of N-heterocyclic fluorinated compounds measured at 1.4 T.

    4.3 ^{19}F longitudinal relaxation times.

    4.4 ^{19}F NMR SABRE enhancements as a function of the polarization transfer field.

5. ^{19}F MRI Experimental Results

6. NMR Characterization

7. References

1. Materials and Methods

1.1 Materials

All of the experimental procedures associated with this work were carried out under nitrogen using standard Schlenk techniques. The solvents used were dried using an Innovative Technology anhydrous solvent system, or distilled from an appropriate drying agent under nitrogen. Catalysts were prepared according to literature methods.[1] Deuterated methanol (MeOD) was obtained from Sigma-Aldrich and used as supplied.

The catalyst precursors [Ir(IMes)(COD)Cl] (**1**) and [Ir(IMe)(COD)Cl] (**2**) were employed. These catalysts were synthesized by established procedures. The SABRE experiments used different concentrations of the substrate and catalyst as detailed below. MeOD was used as the solvent in all cases.

NMR samples were prepared in 5 mm and 10 mm diameter NMR tubes fitted with Young's valves. Samples were degassed prior to *p*-H_2 (3 bars) addition. Typical samples reflect the following situations:

i. 5 mM catalyst precursor + 20 mM substrate (making it a 1-fold excess of ligand) in 0.6 ml solvent (5 mm standard NMR tube).

ii. 5 mM catalyst precursor + 100 mM substrate (making it a 17-fold excess of ligand) in 0.6 ml solvent (5 mm standard NMR tube).

iii. 15.5 µM IMes catalyst + 100 mM substrate (making it a 17-fold excess of ligand) in 3.0 ml solvent (10 mm standard NMR tube).

The substrates analysed are presented in Scheme S1.

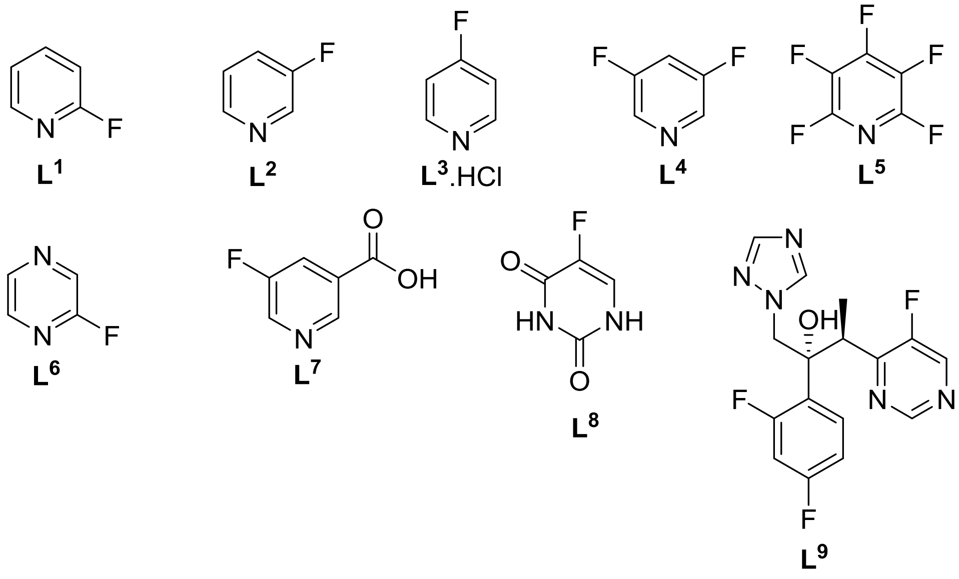

Scheme S1. Substrates analysed in this work.

1.2 Instrumentation and procedures.

Most ^{1}H and all ^{13}C NMR measurements were recorded on a Bruker Avance III series 400 MHz (9.4 T). The sample temperature was 23°C, and the 90° pulse length was 8.5 µs and 15.0 µs for ^{1}H and ^{13}C respectively as determined by the machine's internal calibration cycle. ^{19}F NMR measurements, as well as T_1 inversion recovery experiments were performed on a Bruker Avance III series 500 MHz (11.74 T) system. The pulse length for ^{19}F was 21.5 µs.

Additional ^{1}H and ^{19}F NMR spectra were acquired on a 1.4 T Pulsar low-field spectrometer (Oxford Instruments, Tubney Woods, Abingdon, Oxford, UK, 59.7 MHz 1H frequency and 56.2 MHz ^{19}F frequency) running SpinFlow software (v2.3, Oxford Instruments). The sample temperature was 22°C, and the 90° pulse length was 9.6 µs and 8.4 µs for ^{1}H and ^{19}F respectively as determined by the machine's internal calibration cycle. The linewidth was maintained between 0.5 and 0.9 Hz by daily checking of the chloroform FWHM and shimming as and when necessary.

NMR characterization data was collected at 11.74 T at a temperature of 245 K, using a range of 1-D and 2-D methods that included NOE, COSY and HMQC procedures.[2-6] Some of the resonances could not be identified or assigned due to peak overlap and line broadening. The fast dynamic processes exhibited by the complexes analysed were studied by EXSY methods.[4]

SABRE analysis NMR samples were prepared containing 5 mM catalyst precursor in 0.6 ml of methanol-d_4. Arrays of NMR measurements were collected using either 4 or 20 equivalents of substrate to 5 mM of iridium in 0.6 ml MeOD (leading to samples containing 1- and 17-fold excesses of ligand relative to iridium, respectively). After adding p-H_2 at 3 bar pressure, ^{1}H NMR spectra were recorded using $\pi/2$ excitation pulses immediately after shaking the sample in a magnetic field of 65 G. A similar procedure was used for the polarization of heteronuclei, but the ^{19}F spectra were acquired after shaking the sample in a field of ~ 15 G.

Enhancement factors were calculated by using the ratio of the integral areas of individual resonances in the hyperpolarized spectrum and the spectrum collected under normal H_2 and Boltzmann equilibrium conditions respectively.

Field dependence Polarization transfer field (PTF) measurements were recorded using an automated system that allows for repeated hyperpolarization in the presence of constant low fields of accurate values. Samples containing 10 mg of catalyst precursor (5 mM) and 17-fold excess of ligand respectively were prepared in 3 ml of methanol-d_4. After dissolution, the samples were introduced in the flow system and pulse-and-collect, as well as multi-quantum filtered experiments were performed at PTFs that ranged from 0 to 140 G, in steps of 10 G. The external magnetic field was screened by placing the mixing chamber containing the sample and the PTF coil in a µ-metal shield. Details of the polarizer have been reported.[7]

EXSY measurements and kinetic analysis A series of exchange spectroscopy (EXSY) measurements were made to probe the dynamic behaviour of these systems.[3] This process involved the selective excitation of a single resonance and the subsequent measurement of a ^{1}H NMR spectrum at time, t, after the initial pulse. The resulting measurements consisted of a series of data arrays such that t is varied between 10 and 25 values, typically between 0.1 to 1.0 s, to encode the reaction profile. The precise values were varied with temperature to suit the speed of the process. Data was collected for a range of temperatures and sample concentrations. Integrals for the interchanging peaks in the associated ^{1}H EXSY spectra were obtained and converted into a percentage of the total detected signal.

These data were then analysed as a function of the mixing time according to a differential kinetic model.[8] Rates of exchange were determined by employing a Runge-Kutta[9, 10] scheme to solve the system of differential equations and a Levenberg–Marquardt[11] algorithm to minimize the sum of the residuals in the associated least mean squares analysis. On a case by case basis, the theoretical models used to fit the

experimental EXSY data involved either a two-site exchange (A ↔ B) system, as expressed by the equations below:

$$-\frac{dA}{dt} = -K_{ab} * A + K_{ba} * B$$

$$-\frac{dB}{dt} = +K_{ab} * A - K_{ba} * B$$

or a multi-site exchange system.

An example of typical build-up/decay curves obtained from the integration of the experimental EXSY data, together with the corresponding fitted data is presented in Figure S1. Thermodynamic parameters were calculated using the exchange rates obtained for a wide range of temperatures and the Eyring-Polanyi equation.[12, 13]

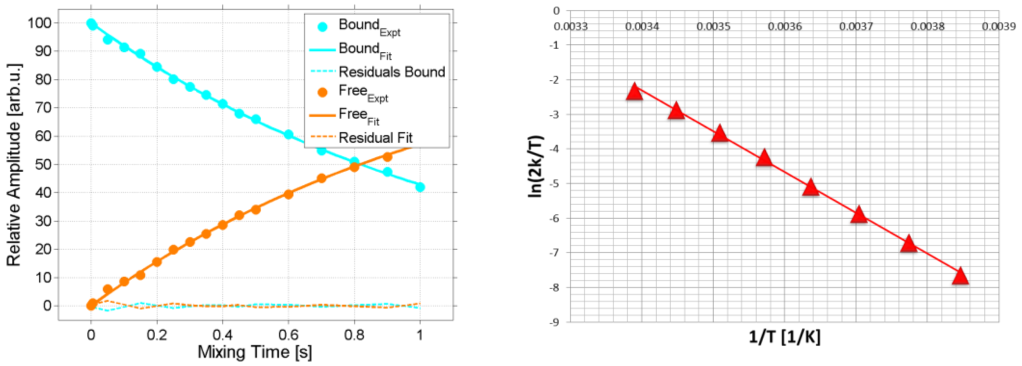

Figure S1. Build-up/decay obtained from the integration of experimental EXSY data recorded for L^2 at 275 K (left) and corresponding Eyring plot for the ligand build-up in solution (right).

2. ^{1}H NMR Experimental Results

2.1. ^{1}H NMR SABRE polarization of fluorinated compounds.

Samples containing 5 mM concentration of catalyst precursor were prepared with a 1-fold excess of substrate and a 17-fold excess of substrate in 600 µl MeOD and were hyperpolarized as described previously in the experimental section. Up to 7 experiments were performed for each sample and an average enhancement factor was calculated.

L^1 has been hyperpolarized under SABRE conditions with an average total enhancement of -1.85 obtained for the sample prepared with 1–fold excess of ligand and -1.15 for the sample prepared with 17-fold excess of ligand. The spectra obtained for the latter are presented in Figure S2.

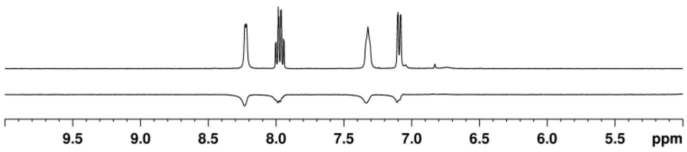

Figure S2. ^{1}H NMR spectra of L^1 (17-fold excess) acquired in Boltzmann equilibrium conditions (top, x 128) and hyperpolarized under SABRE (bottom).

L^2 has been hyperpolarized under SABRE conditions with an average total enhancement of -3562 obtained for the sample prepared with 1–fold excess of ligand and -2867 for the sample prepared with 17-fold excess of ligand. The spectra obtained for the latter are presented in Figure S3.

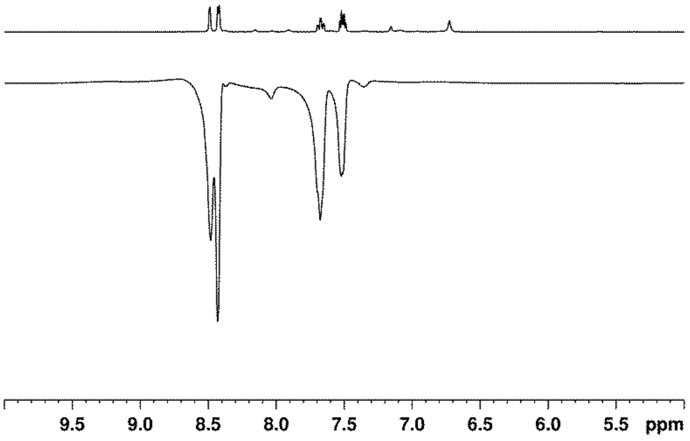

Figure S3. **^{1}H NMR spectra of L^2 (17-fold excess) acquired in Boltzmann equilibrium conditions (top, x 128) and hyperpolarized under SABRE (bottom).**

L^3 is unstable as such and typically provided in the form of a 4-fluoropyridine·HCl formulation. The presence of acid is expected to hinder the binding process. Our attempts at isolating the 4-fluoropyridine permanently using either filtering through a column or addition of a base (Cs_2CO_3 and KOH) have been unsuccessful and no SABRE polarization transfer catalyst could be formed using this substrate in its pure form. However, addition of Cs_2CO_3 proved to be effective in terms of removing the HCl and isolating L^3 temporarily which led to 4-fluoropyridine to react with itself and form N-(4'-pyridyl)-4-fluoropyridinium fluoride. This compound is able to react with **1** and, in the presence of *p*-H_2, to form a SABRE polarization transfer catalyst. However, we found that at low ligand loadings (1-fold excess of substrate and 1-fold excess of base relative to **1**) no enhancement of the proton resonances of either the free or bound material can be detected. We assume that this is due to very rapid longitudinal relaxation.

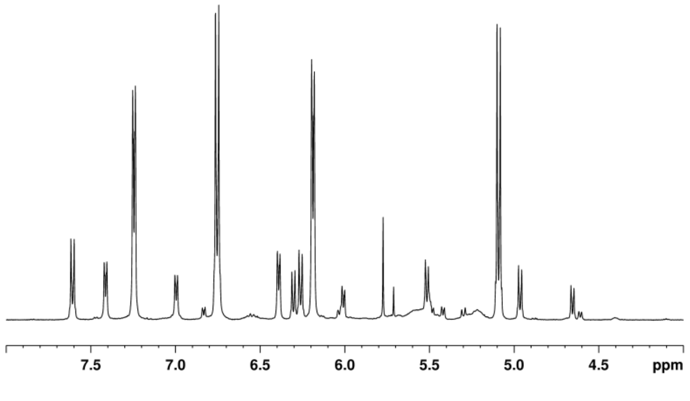

Figure S4. **^{1}H NMR spectrum of L^3.HCl (17-fold excess of ligand and base respectively to 5 mM of 1) acquired under Boltzmann equilibrium conditions using 128 scans.**

When working at high ligand loadings (17-fold excess of substrate and 17-fold excess of base relative to **1**) the substrate's resonances (particularly the ones corresponding to the bound peaks) appear as enhanced; due to significant peak overlap, we have calculated just the average enhancement (corresponding to the sum of the free and bound resonances) and found it to be approximately -22.8. No enhancement could be observed for the resonances belonging to the hydrides. Based on the hypothesis that the presence of Cs_2CO_3 promotes rapid

H-D exchange, we have reduced the amount of base added to the solution to 7-fold excess relative to **1**, and found that the average enhancement increases to -58.8 (Figure S5) and numerous enhanced hydride resonances could be detected in the hyperpolarized spectra (Figure S6).

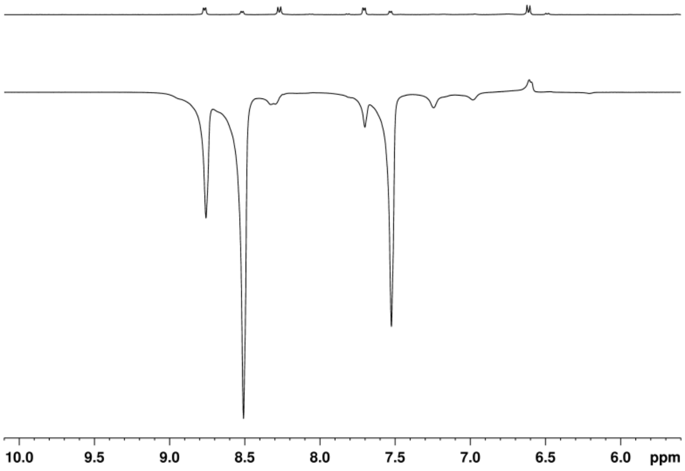

Figure S5. ^{1}H **NMR spectra of** L^{3}**.HCl in Boltzmann equilibrium conditions (top) and hyperpolarized under SABRE (bottom).**

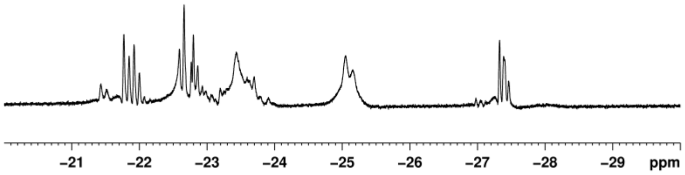

Figure S6. ^{1}H **NMR spectrum of** L^{3}**.HCl in Boltzmann equilibrium conditions (top) and hyperpolarized under SABRE (bottom).**

$\mathbf{L^4}$ has been hyperpolarised under SABRE conditions with an average total enhancement of -1093 obtained for the sample prepared with 1–fold excess of ligand and -795 for the sample prepared with 17-fold excess of ligand. The spectra obtained for the latter are presented in Figure S7.

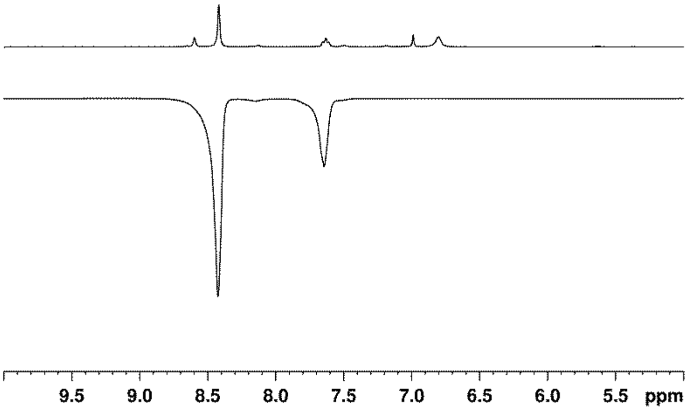

Figure S7. ^{1}H **NMR spectra of** L^{4} **acquired in Boltzmann equilibrium conditions (top, x 128) and hyperpolarized under SABRE (bottom).**

L[6] has been hyperpolarized under SABRE conditions with an average total enhancement of -1176 obtained for the sample prepared with 1–fold excess of ligand and -1087 for the sample prepared with 17-fold excess of ligand. The spectra obtained for the latter are presented in Figure S8.

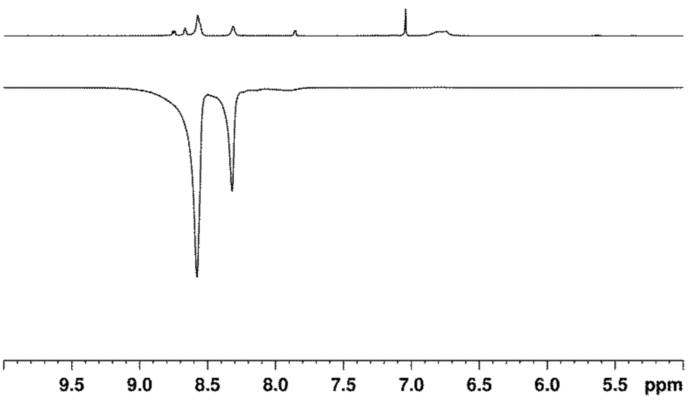

Figure S8. ^{1}**H NMR spectra of L**[6] **acquired in Boltzmann equilibrium conditions (top, x 128) and hyperpolarized under SABRE (bottom).**

2.2 ^{1}H NMR SABRE polarization of biologically relevant fluorinated compounds by pH manipulation.

It is widely known that pyridine carboxylic acids can be present in solution in the form of multiple species, due to their capacity to undergo various protonation events (see Scheme S2, neglecting the dication). In acidic solutions the conjugate acid (a) predominates, whilst in basic environments the conjugate base (d) is essentially the only form present. These forms co-exist at intermediate acidities.[14] As such, we expect that in the presence of **1** in MeOD, 5-fluoroniacin (**L**[7]) can form several polarization transfer catalysts, as a function of the pH of the solution.

We have shown in our previous work[15] that the efficiency of the magnetization transfer, as well as the relaxation and exchange rates, can be modified by increasing the pH of the solution. Addition of a relatively mild base, such as Cs_2CO_3, leads to the deprotonation of the substrate, which improves the probability of the N centre binding to the Ir and thus increases the polarization transfer efficiency. Furthermore, the transition from an acidic to a basic environment has been found to increase the relaxation time of the H-2 resonance.[15]

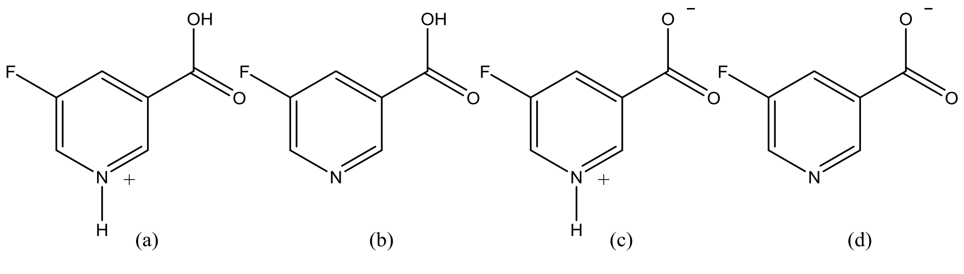

Scheme S2. Possible forms of L[7] **in solution.**

A series of one-shot ^{1}H NMR spectra were collected on samples containing 1-fold and 17-fold excess **L**[7] in the presence of 5 mM of **1** in MeOD solution, using π/2 read-out pulses. In the case of the sample containing 1-fold excess substantial signal enhancements were observed for all three of the non-exchangeable resonances of free substrate, with the largest ^{1}H NMR signal enhancement being observed for H-2 (-272 ± 24), followed by H-4 and H-6 (-181 ± 16 and -121 ± 9 at 400 MHz). The corresponding values for the solution containing 17-fold excess of ligand were 59 ± 2, 50 ± 2 and 55 ± 2.

As shown in the literature,[15-17] methanol can be an active participant in the polarization transfer catalyst and, when employing mild acidic conditions, solvent polarization can be observed and quantified by measuring the enhancement of the O**H** resonance. Evidence of methanol binding to the Ir centre can be obtained by examining the hydride region of the spectra, which in the case of $\mathbf{L^7}$, contains a resonance at δ -23.45, corresponding to the trisubstituted complex and a pair of resonances located at δ -23.84 and -24.04, corresponding to polarization transfer catalysts in which the equatorial sites are occupied by one substrate molecule and one methanol molecule positioned *cis* and *trans* respectively in reference to the carbine. The possibility of the hydride pair belonging to a complex in which Cl binding occurs was excluded by acquiring spectra of the same complex dissolved in dichloromethane (DCM) and comparing the results. ^{1}H SABRE NMR experiments show that significant methanol binding occurs for both samples prepared with $\mathbf{L^7}$ and the O**H** resonance is polarised in both cases, exhibiting average enhancement values of 6.65 ± 0.5 (1-fold excess) and 51 ± 2 (17-fold excess).

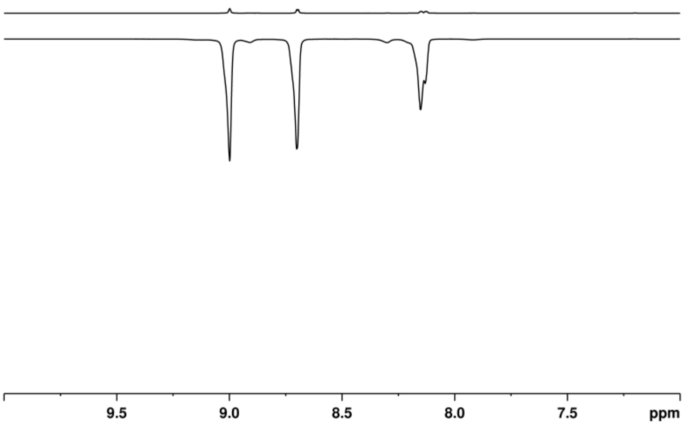

a).

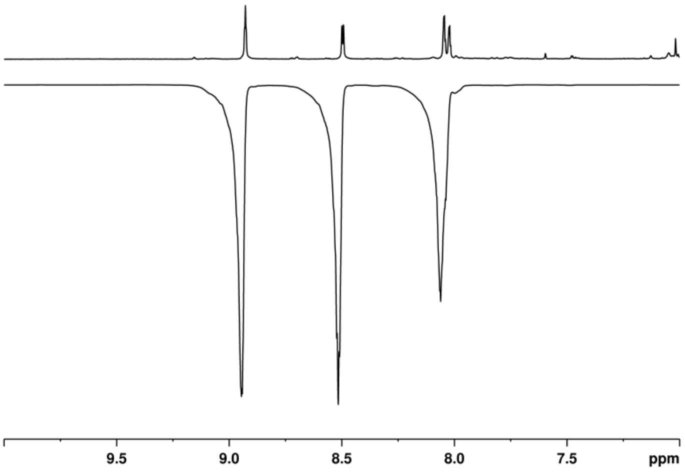

b).

Figure S9. ^{1}H NMR spectra of L^7acquired in Boltzmann equilibrium conditions and hyperpolarized under SABRE. a). 17-fold excess ligand ($L^7{}_a$), b). 17-fold excess ligand and 17-fold excess Cs_2CO_3 ($L^7{}_b$). For comparison purposes the spectra have been plotted on the same scale.

When analysing the same samples after adding Cs_2CO_3 in equal amounts to the substrate (1-fold and 17-fold excess respectively), we found that the enhancement of the free ligand resonances increases considerably in both cases, as a result of the base deprotonating the ligand and making it more suitable for binding to the Ir centre (Figure S9). We note that in the case of low ligand loading, the presence of base promotes and accelerates H-D exchange, a phenomenon which occurs immediately after activation and on the time scale of the experiments, resulting in a progressive decrease of the total

enhancement with each addition of fresh *p*-H_2 (see Table S1). Data show that ~ 40% deuteration of the substrate takes place in the first 15 minutes after activation (Figure S10).

Table S1. Decrease in enhancement due to H-D exchange.

	H-2	H-4	H-6
1st experiment	-809	-246	-352
2nd experiment	-627	-189	-274
3rd experiment	-470	-137	-208

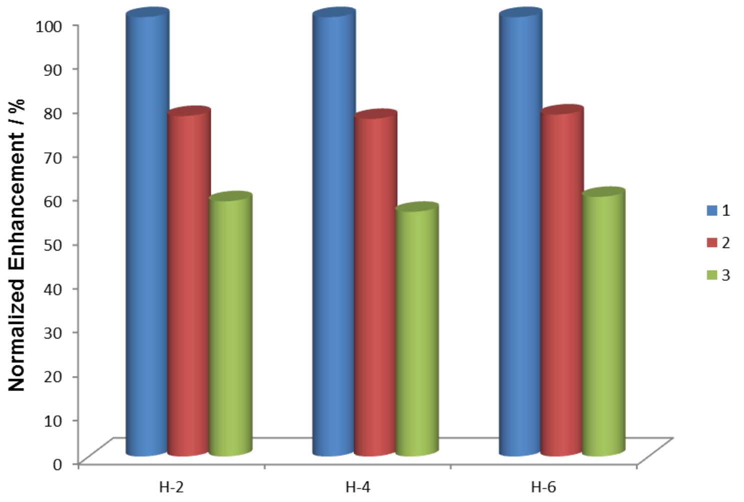

Figure S10. Decrease in enhancement due to H-D exchange. The values have been normalized to the enhancement obtained in the first experiment, performed immediately after catalyst activation.

For the sample prepared using 1-fold excess of ligand and 1-fold excess of base relative to the catalyst, the total maximum enhancement obtained is 2.5 times higher than for the similar sample prepared without base; the corresponding enhancement for the high (17-fold) ligand loading sample increased 4.5 times. Furthermore, no methanol binding and O**H** enhancements could be observed in this situation. These results are in agreement with our previous work[15] which shows the beneficial effect of pH manipulation has on the efficiency of substrate binding to the Ir centre.

We have adopted a similar strategy for improving the enhancement in the case of $\mathbf{L^8}$, as this molecule has both N atoms protonated, a fact that prevents the ligand binding efficiently to the catalyst and hinders activation. We have tried to promote the activation process by adding acetonitrile to the sample, which led to the catalyst being activated but the enhancement exhibited by the free ligand resonance was below 1 (Figure S11).

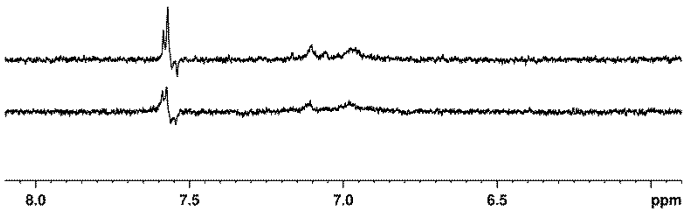

Figure S11. **^{1}H NMR spectra of L^{8} acquired in Boltzmann equilibrium conditions (top) and hyperpolarized under SABRE in the presence of acetonitrile (bottom).**

As the protonation of the N atoms is known to hinder binding, we have added a base (Cs_2CO_3) to **L^8** with the purpose of deprotonating the N centres and easing the process of binding (Scheme S3).

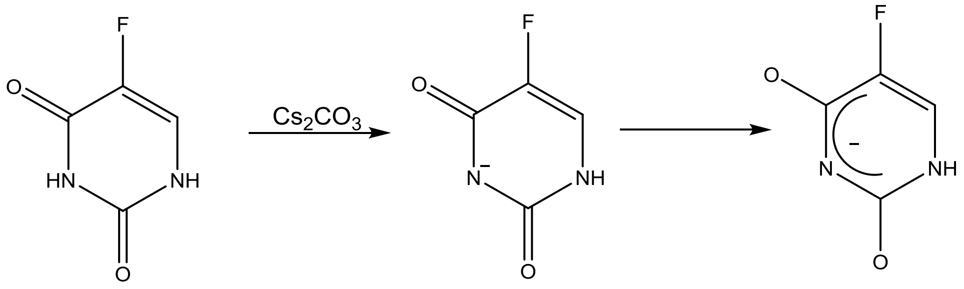

Scheme S3. Deprotonation of L^8 due to addition of Cs_2CO_3.

A sample containing 7-fold excess **L^8** and 3 equivalents of base was analysed under SABRE conditions and an 8.5-fold enhancement was obtained for the free ligand resonance (Figure S13). Unfortunately, a cyclometalation reaction involving the methyl groups of the IMes substituent quenches the SABRE catalyst activity approximately 15 minutes after activation.

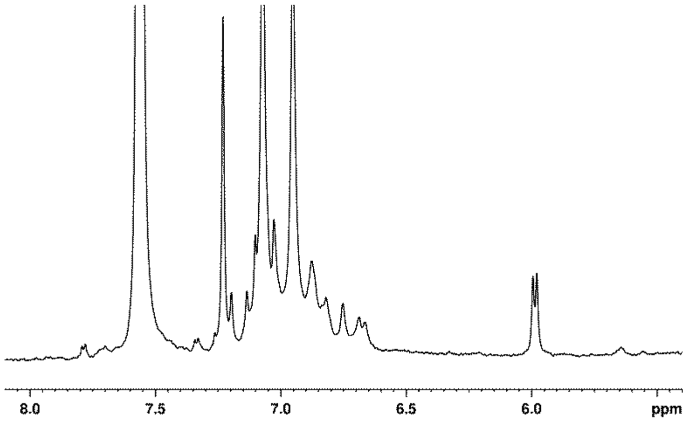

Figure S12. **^{1}H NMR spectrum of L^{8} acquired in Boltzmann equilibrium conditions after catalyst activation. A bound ligand resonance can be detected at ~ 6 ppm.**

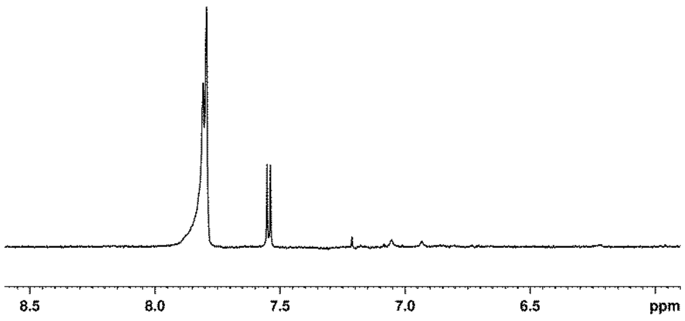

Figure S13. **^{1}H NMR spectrum of L^8 acquired in hyperpolarized under SABRE in the presence of base.**

Higher enhancements have been obtained after changing the substrate and base concentration. A sample containing 5 mM of **1**, 1-fold excess of ligand and 0.5 equivalents of base has been analyzed as a function of time since activation and it was found that immediately after activation an enhancement of -53 can be obtained for the free resonance, followed by a progressive decrease due to the cyclometalation process (Figure S14).

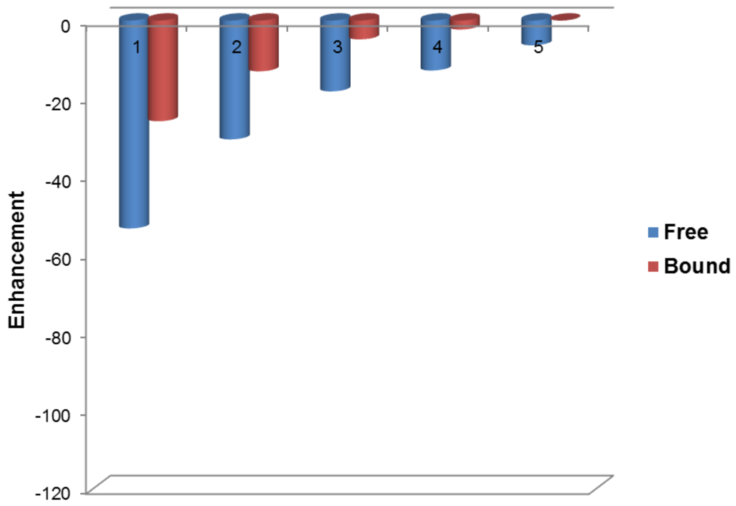

Figure S14. **^{1}H enhancement values for the free resonance of L^8 (1-fold excess, 0.5 equivalents of base) determined from spectra acquired immediately after catalyst activation. The decrease in enhancement as a function of the experiment number shows the development of the cyclometalation process as a function of time.**

Further optimization led to a maximum enhancement of -100 for a sample containing 1-fold excess of ligand and 3 equivalents of base analysed immediately after adding *p*-H_2 (Figure S15).

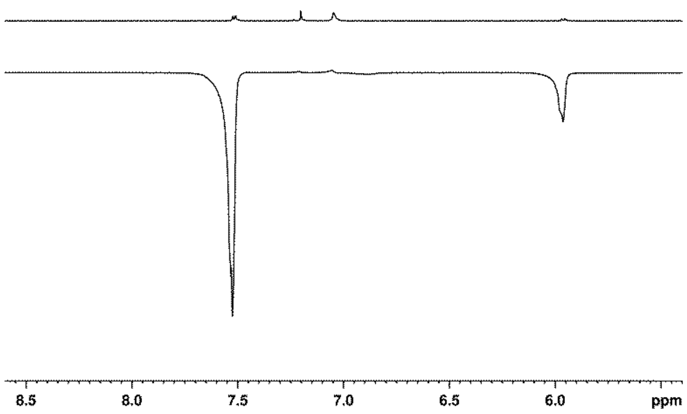

Figure S15. ^{1}**H NMR spectra of L**^{8} **acquired in Boltzmann equilibrium conditions (top) and hyperpolarized under SABRE in the presence of base (bottom) immediately after activation.**

Experiments performed after the activation process show the same gradual decrease in enhancement as a consequence of the cyclometalation process (Figure S16).

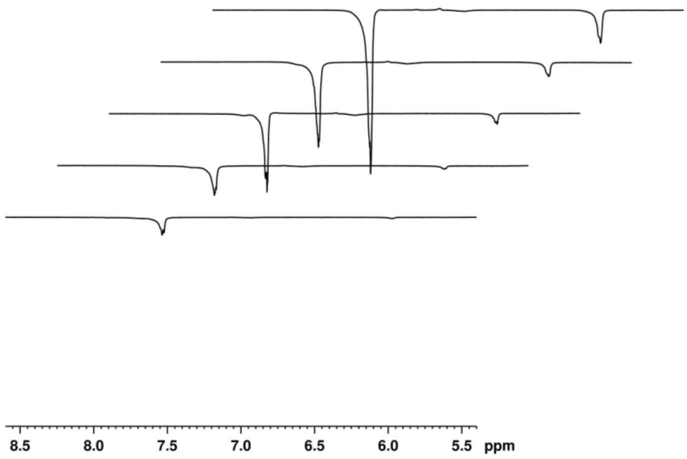

Figure S16. ^{1}**H NMR spectra of L**^{8} **acquired consecutively after activation (first spectrum top of figure, last spectrum bottom)**

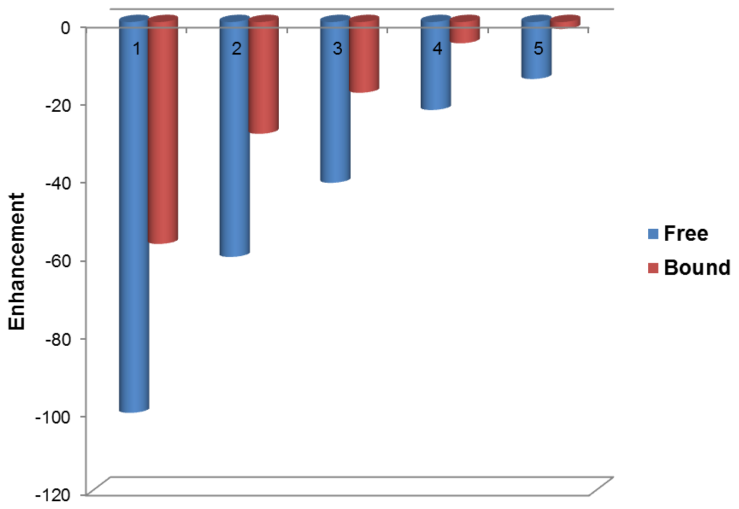

Figure S17. **^{1}H NMR enhancements of L^8 (1-fold excess, 2 equivalents of base) acquired immediately after activation. The decrease in enhancement as a function of the experiment number shows the development of the cyclometalation process as a function of time.**

The individual and total enhancements obtained for the substrates tested are presented in Table S2 and S3.

Table S2. **^{1}H NMR SABRE enhancements obtained for the individual protons of the substrates tested.**

	L^1	L^2	L^4	L^6	L^7_a	L^7_b	L^8
**1-fold excess**							
**H2**	-	-1286 ± 145	-591 ± 24	-	-272 ± 24	-809*	-100
**H3**	-0.45 ± 0.12	-	-	-715 ± 84	-	-	-
**H4**	-0.45 ± 0.11	-534 ± 40	-501 ± 27	-	-181 ± 16	-245*	-
**H5**	-0.28 ± 0.10	-924 ± 85	-	-461 ± 39	-	-	-
**H6**	-0.66 ± 0.19	-817 ± 59	-591 ± 24	overlap	-112 ± 9	-352*	-
**17-fold excess**							
**H2**	-	-861 ± 145	-454 ± 26	-	-59 ± 2	-254 ± 22	-
**H3**	-0.28 ± 0.05	-	-	-618 ± 39	-	-	-
**H4**	-0.28 ± 0.04	-364 ± 25	-341 ± 25	-	-50 ± 2	-222 ± 13	-
**H5**	-0.21 ± 0.04	-1207 ± 70	-	-469 ± 26	-	-	-
**H6**	-0.36 ± 0.06	-435 ± 95	-454 ± 26	overlap	-55 ± 2	-247 ± 15	-

*Maximum value obtained. An average enhancement value and the associated error could not be determined due to fast H-D exchange on the substrate molecule.

Table S3. **^{1}H NMR SABRE total enhancements obtained for the substrates tested.**

	L^1	L^2	L^3	L^4	L^6	L^7_a	L^7_b	pyridine[a]
**1-fold excess**	-1.85 ± 0.28	-3562 ± 183	-	-1093 ± 36	-1177 ± 92	-565 ± 34	-1407.13*	5498
**17-fold excess**	-1.14 ± 0.10	-2867 ± 190	-58 ± 5[b]	-795 ± 36	-1087 ± 47	-162 ± 3	-724 ± 30	907

[a]Magnitude enhancement values, as reported in by Lloyd and coworkers.[18]

[b]After reacting to form N-(4'-pyridyl)-4-fluoropyridinium fluoride.

When comparing the results obtained on the fluorinated compounds studied in this work with the ones obtained by Lloyd et al,[18] we find that, when working at low ligand loadings, the total enhancement obtained for each substrate is considerably lower than for pyridine. However, when moving on to high ligand loadings, the performance of pyridine is surpassed by $\mathbf{L^2}$ and $\mathbf{L^6}$ (with enhancements which are 3.2 and 1.2 higher) while the enhancements obtained for $\mathbf{L^4}$ and $\mathbf{L^7_b}$ become comparable to the ones of pyridine. This can be explained by factoring in the effect of relaxation and exchange rates on the polarization transfer, as it will be shown in the following sections.

2.3 ^{1}H longitudinal relaxation times.

Longitudinal relaxation times (T_1) have been determined experimentally using the inversion recovery method for samples containing 17-fold excess of $\mathbf{L^2}$, $\mathbf{L^4}$, $\mathbf{L^6}$, $\mathbf{L^7_a}$ and $\mathbf{L^7_b}$. The results are presented in Table S4, together with the corresponding values for pyridine measured in the same conditions, as reported by Lloyd and coworkers.[18]

Table S4. ^{1}H longitudinal relaxation times of the individual protons of the substrates tested.

	$\mathbf{L^2}$	$\mathbf{L^4}$	$\mathbf{L^6}$	$\mathbf{L^7_a}$	$\mathbf{L^7_b}$	**pyridine***
**17-fold excess**						
**H2**	19.03	21.90	-	14.52	46.14	12.6
**H3**	-	-	22.59	-	-	14.7
**H4**	13.47	29.24	-	14.54	16.04	18.9
**H5**	21.11	-	25.28	-	-	14.7
**H6**	11.56	21.90	27.09	12.73	16.91	12.6

*Taken from [18].

The data show that all substrates analyzed exhibit relatively long relaxation times, higher or comparable to those of pyridine. The lowest values measured correspond to 5-fluoronicotinic acid in its protonated form ($\mathbf{L^7_a}$), probably due to the presence of a hydrogen atom on the binding centre. As shown in our previous work,[15] addition of base and the subsequent change in pH has a favourable effect on the T_1 of H-2 which, in this case, increases remarkably from 14.5 to 46.1 s. Smaller increases can also be noted for protons H-4 and H-6.

2.4 Exchange rates measured as a function of temperature.

A series of exchange spectroscopy (EXSY) measurements were made to probe the dynamic behaviour of the polarization transfer catalyst formed by the ligands investigated in this work with **1** after H_2 addition. This process involved the selective excitation of a single resonance and the subsequent measurement of a ^1H NMR spectrum at time, *t*, after the initial pulse. The resulting measurements consisted of a series of data arrays such that *t* is varied between 10 and 25 values, typically between 0.1 to 1.0 s, to encode the reaction profile. The precise values were varied with temperature to suit the speed of the process. Data was collected for a range of temperatures and sample concentrations. Integrals for the interchanging peaks in the associated ^1H EXSY spectra were obtained and converted into a percentage of the total detected signal. When possible, we have measured the exchange rates and activation parameters for both catalyst species present in solution, i.e. $[Ir(IMes)(H)_2(\mathbf{L})_3]Cl$ and $[Ir(IMes)(H)_2(\mathbf{L})_2(MeOD)]Cl$. In some cases the exchange rates and activation parameters could not be determined either due to hydride deuteration taking place on the time scale of the experiments (leading to poor SNR) or due to the complexity of the exchange process caused by the presence of two catalyst species in solution.

Table S5. Ligand build-up rates as a function of temperature for $[Ir(IMes)(H)_2(L^2)_3]Cl$.

Temperature / K	*k* / s^{-1}
260	0.061 ± 0.002
265	0.162 ± 0.002
270	0.382 ± 0.008
275	0.847 ± 0.004
280	2.047 ± 0.010
285	4.190 ± 0.010
290	8.226 ± 0.080
295	14.457 ± 0.186

Table S6. Activation parameters for L^2 build-up in solution for $[Ir(IMes)(H)_2(L^2)_3]Cl$.

Activation Parameters	3-fluoropyridine
$\Delta H^{\neq}$ (kJ mol^{-1})	98.0
+/-	4.0
$\Delta S^{\neq}$ (J K^{-1} mol^{-1})	116.6
+/-	14.4
$\Delta G^{\neq}_{300}$ (kJ mol^{-1})	63.0
+/-	1
R Square	0.998

Table S7. Ligand build-up rates as a function of temperature for $[Ir(IMes)(H)_2(L^6)_3]Cl$.

Temperature / K	*k* / s^{-1}
260	0.025 ± 0.001
265	0.063 ± 0.001
270	0.181 ± 0.002
275	0.434 ± 0.003
280	0.993 ± 0.008
285	1.597 ± 0.023
290	2.341 ± 0.039
295	3.254 ± 0.037

Table S8. Activation parameters for L^6 build-up in solution for $[Ir(IMes)(H)_2(L^6)_3]Cl$.

Activation Parameters	2-fluoropyrazine
$\Delta H^{\neq}$ (kJ mol^{-1})	88.3
+/-	14.6
$\Delta S^{\neq}$ (J K^{-1} mol^{-1})	73.6
+/-	52.7
$\Delta G^{\neq}_{300}$ (kJ mol^{-1})	66.2
+/-	1
R Square	0.973

Table S9. Ligand build-up rates as a function of temperature for $[Ir(IMes)(H)_2(L^6)_2(MeOD)]Cl$.

Temperature / K	k / s^{-1}
255	0.061 ± 0.001
260	0.181 ± 0.002
265	0.406 ± 0.007
270	0.704 ± 0.006
275	1.098 ± 0.048
280	1.826 ± 0.030

Table S10. Activation parameters for L^6 build-up in solution for $[Ir(IMes)(H)_2(L^6)_2(MeOD)]Cl$.

Activation Parameters	2-fluoropyrazine
$\Delta H^{\neq}$ (kJ mol^{-1})	76.1
+/-	17.6
$\Delta S^{\neq}$ (J K^{-1} mol^{-1})	39.6
+/-	65.9
$\Delta G^{\neq}_{300}$ (kJ mol^{-1})	64.2
+/-	1
R Square	0.973

Table S11. Ligand build-up rates as a function of temperature for $[Ir(IMes)(H)_2(L^7{}_b)_3]Cl$.

Temperature / K	k / s^{-1}
260	0.019 ± 0.001
265	0.052 ± 0.001
270	0.111 ± 0.001
275	0.265 ± 0.003
280	0.556 ± 0.005
285	0.974 ± 0.002

Table S12. Activation parameters for L^7_b build-up in solution for $[Ir(IMes)(H)_2(L^7_b)_3]Cl$.

Activation Parameters	5-fluoronicotinic acid (basic conditions)
$\Delta H^{\neq}$ (kJ mol^{-1})	94.8
+/-	7.4
$\Delta S^{\neq}$ (J K^{-1} mol^{-1})	94.8
+/-	27.4
$\Delta G^{\neq}_{300}$ (kJ mol^{-1})	66.4
+/-	1
R Square	0.996

Table S13. Hydrogen dissociation rates as a function of temperature for $[Ir(IMes)(H)_2(L^7_b)_3]Cl$.

Temperature / K	*k* / s^{-1}
270	0.04 ± 0.001
275	0.12 ± 0.003
280	0.34 ± 0.005
285	0.58 ± 0.005
290	1.13 ± 0.005
295	2.04 ± 0.009
300	3.92 ± 0.059

Table S14. Activation parameters for hydrogen loss for $[Ir(IMes)(H)_2(L^7_b)_3]Cl$.

Activation Parameters	H_2
$\Delta H^{\neq}$ (kJ mol^{-1})	96.7
+/-	11.5
$\Delta S^{\neq}$ (J K^{-1} mol^{-1})	95.2
+/-	40.4
$\Delta G^{\neq}_{300}$ (kJ mol^{-1})	68.1
+/-	1
R Square	0.989

2.6 ^{1}H NMR SABRE enhancements as a function of the polarization transfer field.

Experiments have been performed using an automated device equipped with a pH_2 delivery system which continuously delivers gas into the polariser which is subsequently transferred into a mixing chamber that contains the sample in solution. The gas is bubbled through the liquid for a defined time in the presence of a coil that produces the local magnetic field required for the polarization process to take place. After magnetization transfer occurs, the sample is delivered by the polariser to the high field magnet for detection.[7]

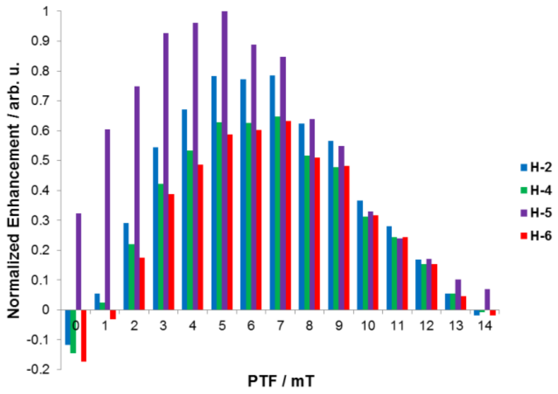

Figure S18. Normalized SABRE ^{1}H individual enhancements for the free L^{2} resonances.

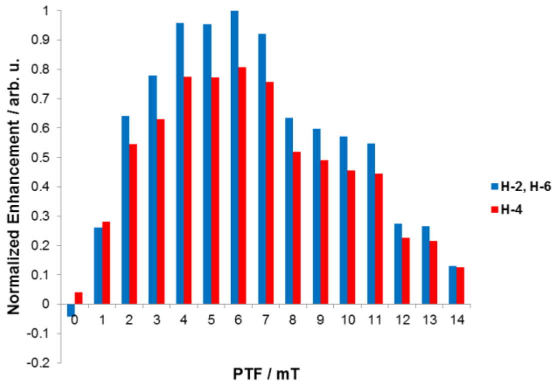

Figure S19. Normalized SABRE ^{1}H individual enhancements for the free L^{4} resonances.

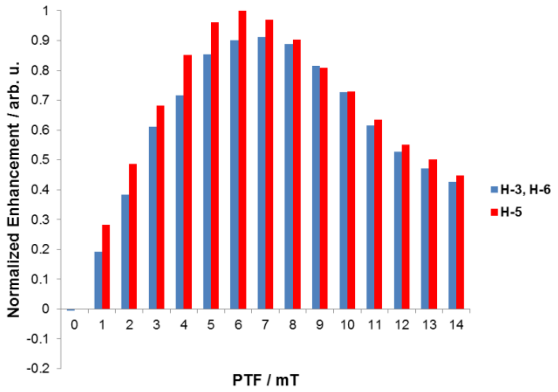

Figure S20. Normalized SABRE ^{1}H individual enhancements for the free L^{6} resonances.

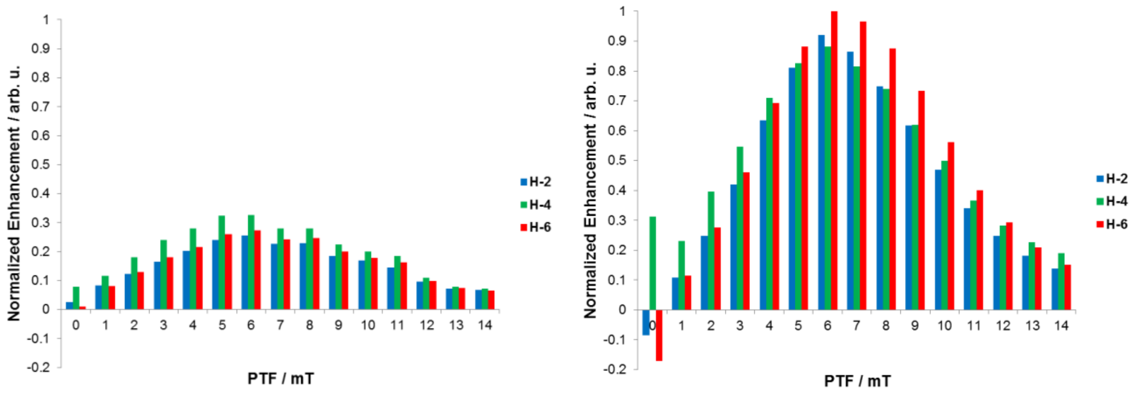

Figure S21. Normalized SABRE ^{1}H individual enhancements for the L^{7} resonances in acidic (left) and basic (right) conditions. For comparison purposes, data have been normalized to the maximum enhancement value obtained (H-6 in the presence of base) and plotted on the same scale.

3. ^{13}C NMR Experimental Results

^{13}C hyperpolarized NMR spectra of the ligands analysed in this work were acquired using INEPT (Insensitive Nuclei Enhanced by Polarization Transfer).[19] The combination between non-selective transfer of polarization from ^{1}H to ^{13}C and SABRE hyperpolarization has allowed us to record high-quality ^{13}C spectra in less than one second. The results obtained are presented below, together with reference spectra, acquired in Boltzmann equilibrium conditions using 64 scans.

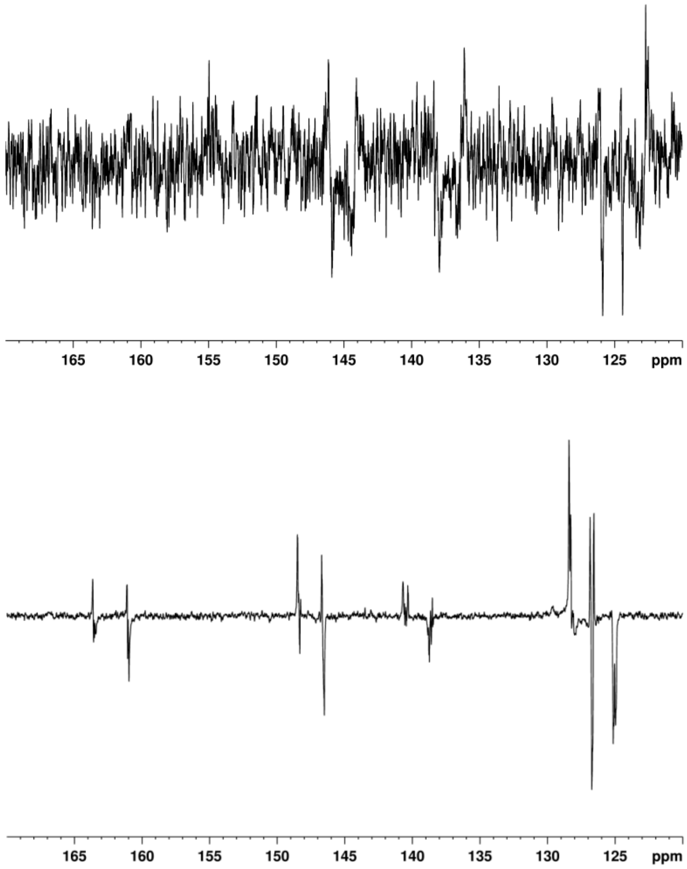

Figure S22. ^{13}**C INEPT spectrum of L^{2} (17-fold excess to 5 mM of 1). Top: Boltzmann equilibrium conditions, 64 averages. Bottom: Hyperpolarized under SABRE, 1 average.**

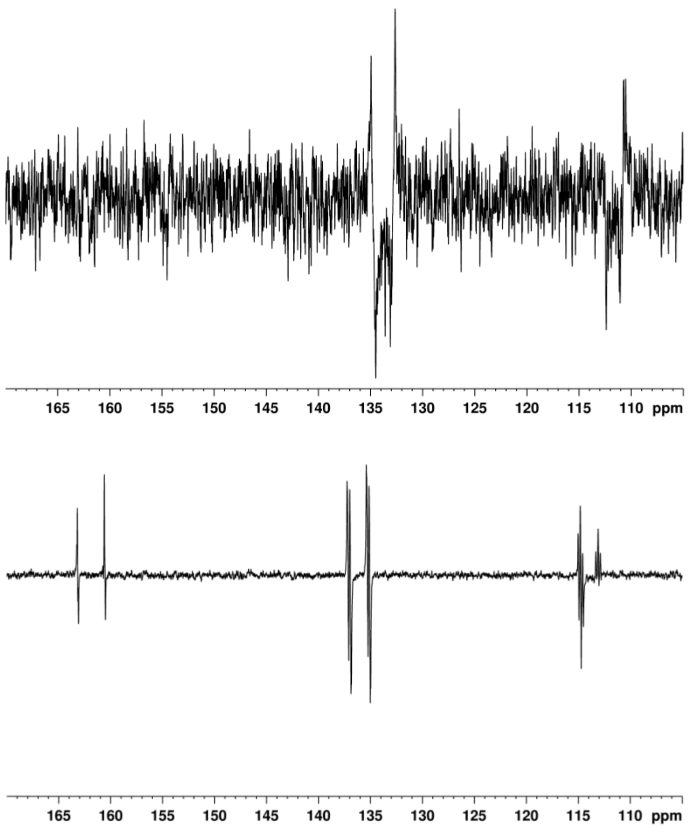

Figure S23. ^{13}C INEPT spectrum of L^4 (17-fold excess to 5 mM of 1). Top: Boltzmann equilibrium conditions, 64 averages. Bottom: Hyperpolarized under SABRE, 1 average.

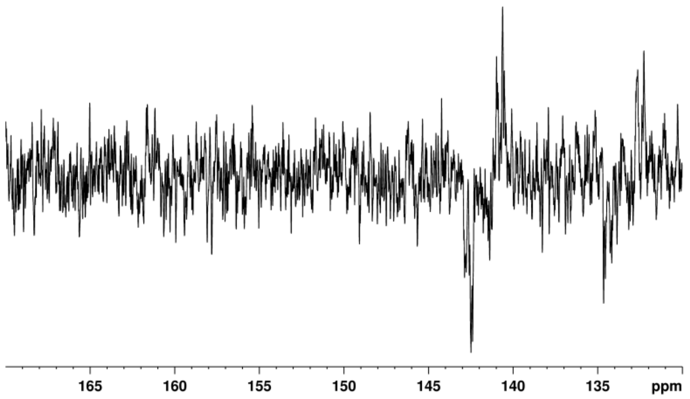

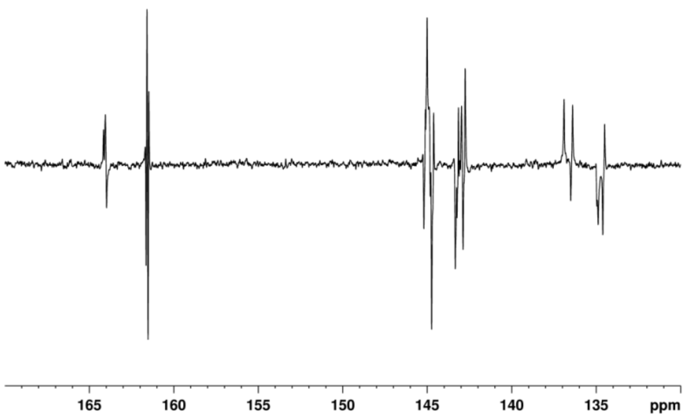

Figure S24. ^{13}**C INEPT spectrum of L**^{6} **(17-fold excess to 5 mM of 1). Top: Boltzmann equilibrium conditions, 64 averages. Bottom: Hyperpolarized under SABRE, 1 average.**

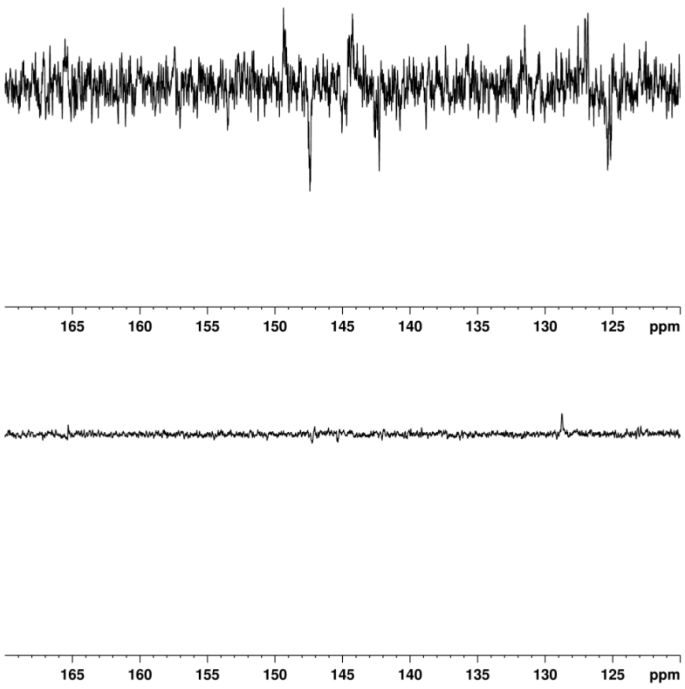

Figure S25. ^{13}**C INEPT spectrum of L**$^{7}_{a}$ **(17-fold excess to 5 mM of 1). Top: Boltzmann equilibrium conditions, 64 averages. Bottom: Hyperpolarized under SABRE, 1 average.**

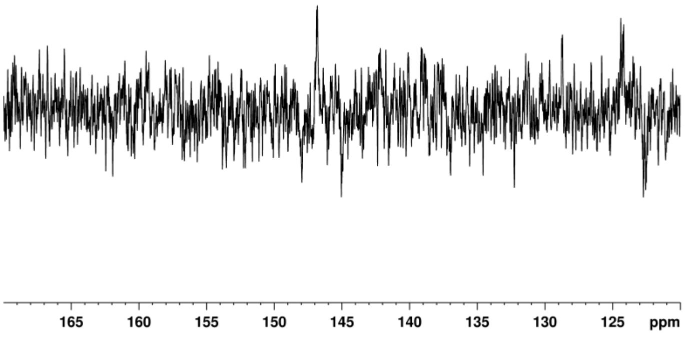

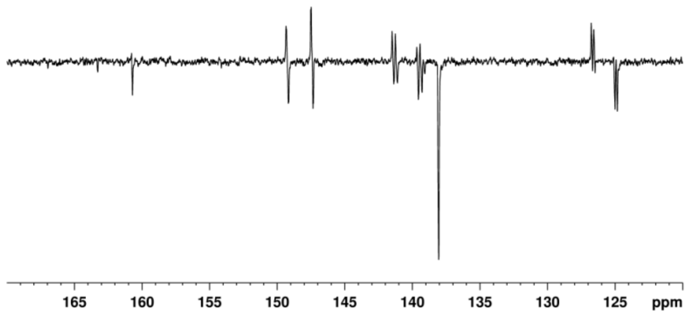

Figure S26. ^{13}C INEPT spectrum of L^7_b (17-fold excess of ligand and 17-fold excess of Cs_2CO_3 to 5 mM of 1). Top: Boltzmann equilibrium conditions, 64 averages. Bottom: Hyperpolarized under SABRE, 1 average.

4. ^{19}F NMR Experimental Results

4.1 ^{19}F NMR SABRE polarization of N-heterocyclic fluorinated compounds measured at 11.7 T.

^{19}F NMR SABRE hyperpolarized spectra of the substrates studied in this work were recorded using a 500 MHz (11.74) Avance III Bruker spectrometer equipped with a triple resonance high-resolution (TBO) 5 mm probe. After addition of *p*-H_2, the samples were shaken in the stray field of the magnet and 90° spectra were collected using one scan. The results obtained are presented below, together with reference spectra, acquired in Boltzmann equilibrium conditions (NS=1).

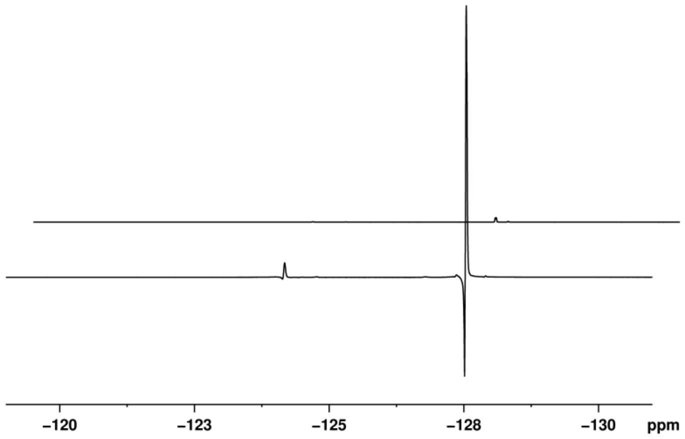

Figure S27. **^{19}F NMR spectra of L^2 (17-fold excess) acquired in Boltzmann equilibrium conditions and hyperpolarized under SABRE.**

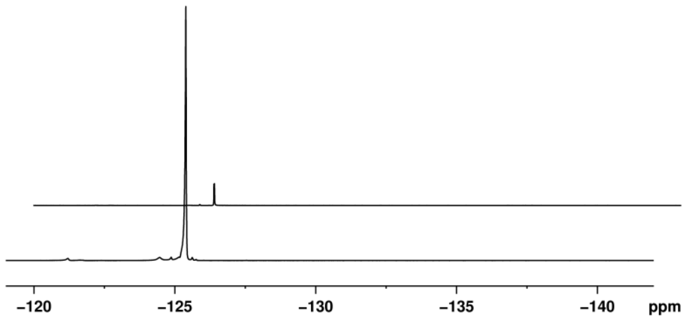

Figure S28. **^{19}F NMR spectra of L^4 (17-fold excess) acquired in Boltzmann equilibrium conditions and hyperpolarized under SABRE.**

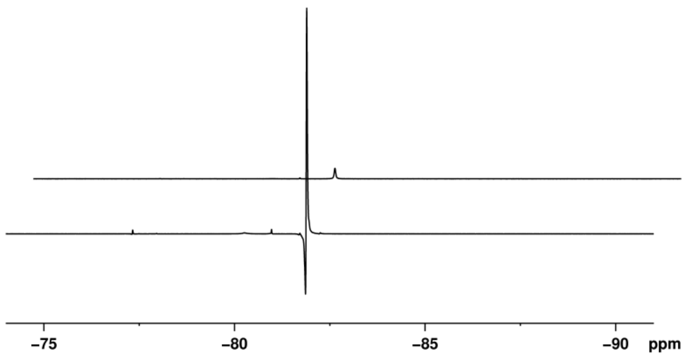

Figure S29. **^{19}F NMR spectra of L^6 (17-fold excess) acquired in thermal equilibrium conditions and hyperpolarized under SABRE.**

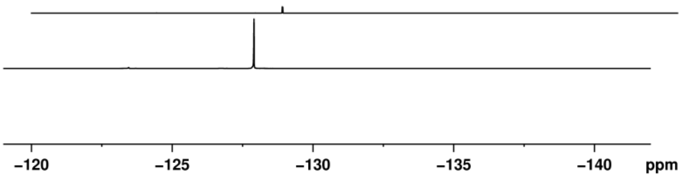

a).

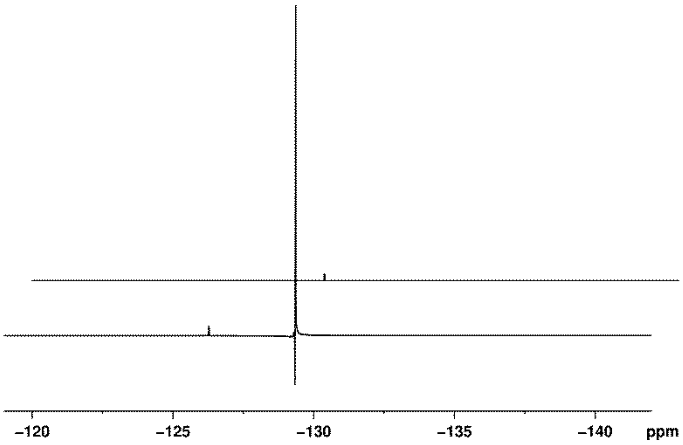

b).

Figure S30. ^{19}F NMR spectra of L^7 acquired in Boltzmann equilibrium conditions and hyperpolarized under SABRE. a). 17-fold excess ligand (L^7_a), b). 17-fold excess ligand and 17-fold excess Cs_2CO_3 (L^7_b). For comparison purposes the spectra have been plotted on the same scale.

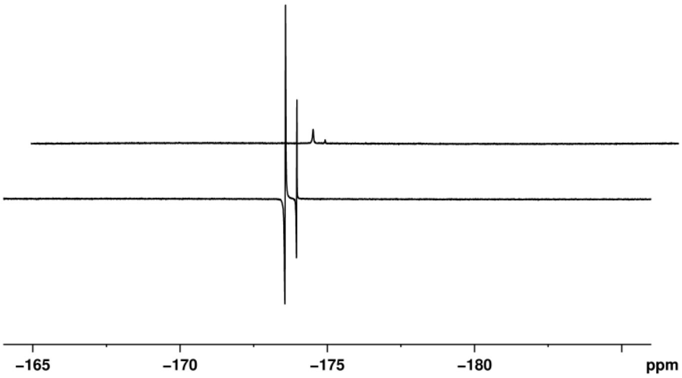

Figure S31. ^{19}F NMR spectra of L^8 (7-fold excess) acquired in Boltzmann equilibrium conditions and hyperpolarized under SABRE.

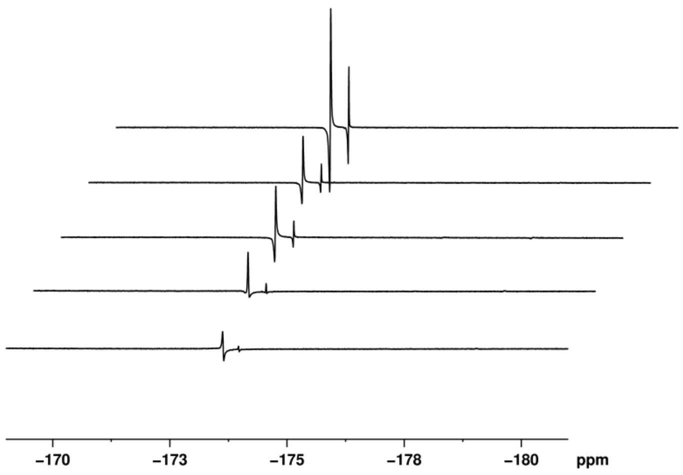

Figure S32. ^{19}F NMR spectra of hyperpolarized L^8 versus time. The enhancement decreases due to cyclometalation, allowing for an online monitoring of the reaction.

Table S15. ^{19}F NMR enhancements obtained using pulse-and-collect experiments.

Ligand Excess	L^2	L^4	L^6	L^7_a	L^7_b
1-fold	61.7 ± 5.8	14.3 ± 1.3	12.2 ± 2.2	50	4.5 ± 1.4
17-fold	60.5 ± 8.5	11.7 ± 1.5	12.1 ± 1.8	8.7 ± 0.9	38.9 ± 3.4

Table S16. ^{19}F NMR enhancements obtained using INEPT experiments.

Ligand Excess	L^2	L^4	L^6	L^7_a	L^7_b
ND	63.7 ± 8.5	15.6 ± 1.5	15.1 ± 2.3	6.2 ± 1.2	98.8 ± 6.4
RD	233.7 ± 22.4	27.7 ± 3.9	17.0 ± 2.2	15.9 ± 07	86.1 ± 3.8

4.2 ^{19}F NMR SABRE polarization of N-heterocyclic fluorinated compounds measured at 1.4 T.

Spectra of compounds studied in this work were acquired at 1.4 T, as described in section 1 except that samples were shaken in earth's magnetic field. The raw time domain data were Fourier-transformed, phase-corrected using SpinFlow and MNova (v9.1, Mestrelab Research, Santiago de Compostela, Spain) software packages to present a single frequency-domain spectrum from each extract. Where spectra were examined qualitatively, apodization (1 Hz exponential) was additionally applied to the FIDs. SABRE-SHEATH measurements utilised a LakeShore cryogenics model 4065 zero gauss chamber to conduct polarisation transfer within.

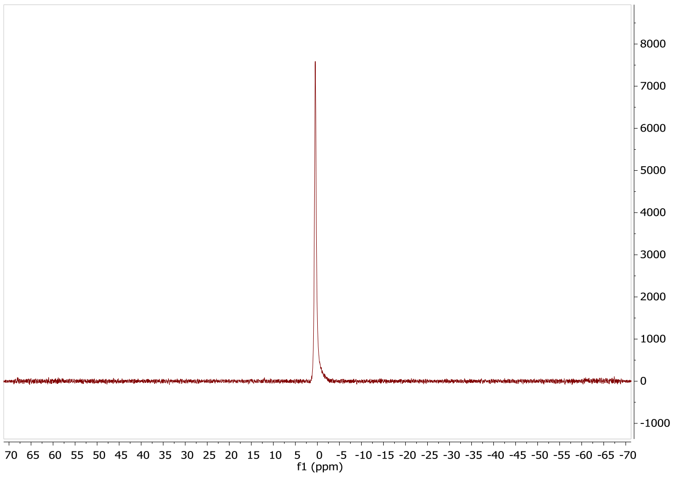

Figure S33. ^{19}**F NMR spectrum of L**^{1} **(17-fold excess to 5 mM of 1 in MeOD) acquired in Boltzmann equilibrium conditions (NS = 256).**

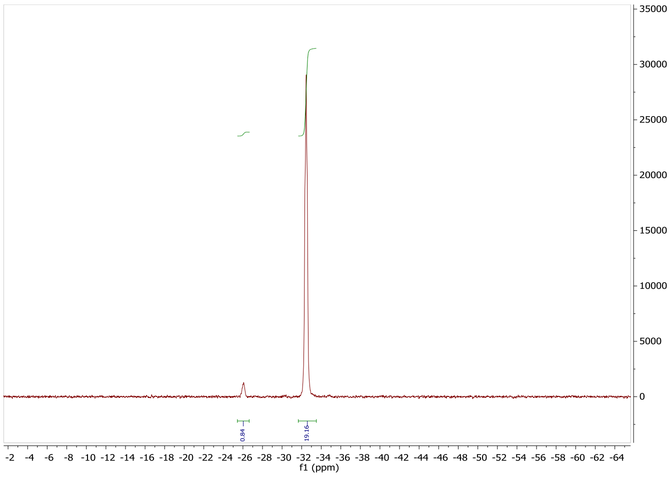

Figure S34. ^{19}**F NMR spectrum of L**^{2} **(17-fold excess to 5 mM of 1 in MeOD) acquired in Boltzmann equilibrium conditions (NS = 256).**

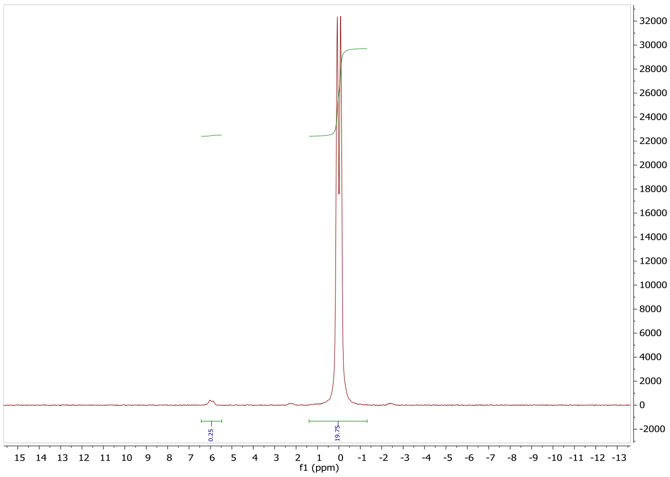

Figure S35. ^{19}**F NMR spectrum of L**^{4} **(17-fold excess to 5 mM of 1 in MeOD) acquired in Boltzmann equilibrium conditions (NS = 256).**

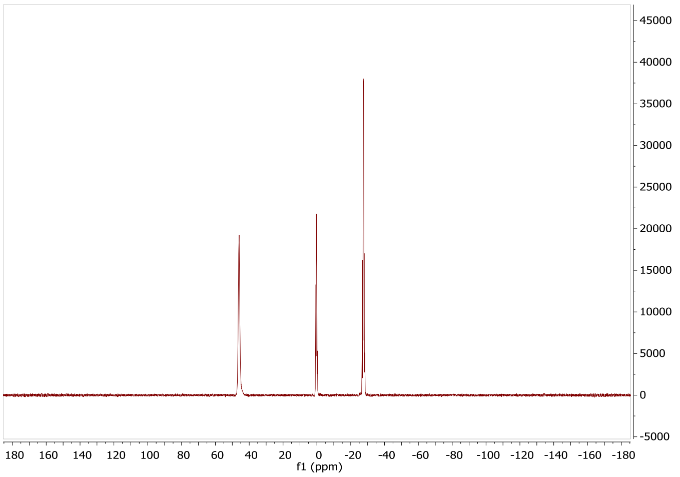

Figure S36. ^{19}F **NMR spectrum of** L^5 **(17-fold excess to 5 mM of 1 in MeOD) acquired in Boltzmann equilibrium conditions (NS = 256).**

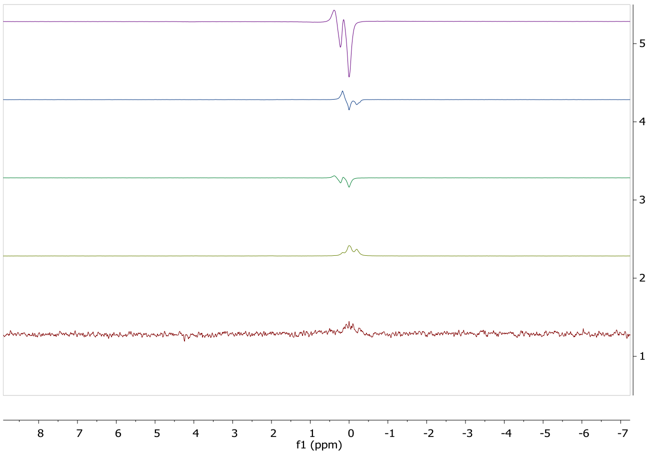

Figure S37. ^{19}F **NMR spectra of a sample consisting of** L^4 **and 1 (1-fold excess of ligand) in MeOD. Spectrum 1: Single scan thermally polarized spectrum (magnified 64 times relative to other spectra); 2: spectrum produced after hyperpolarization in the Earth's magnetic field; 3: spectrum produced after hyperpolarization in a µ-magnetic shield (SABRE-SHEATH); 4: spectrum produced after cooling the sample in an ice bath for 90 s and subsequent hyperpolarization in the Earth's magnetic field; 5: spectrum produced after cooling sample in an ice bath for 90 s and subsequent hyperpolarization in a µ-magnetic shield (SABRE-SHEATH).**

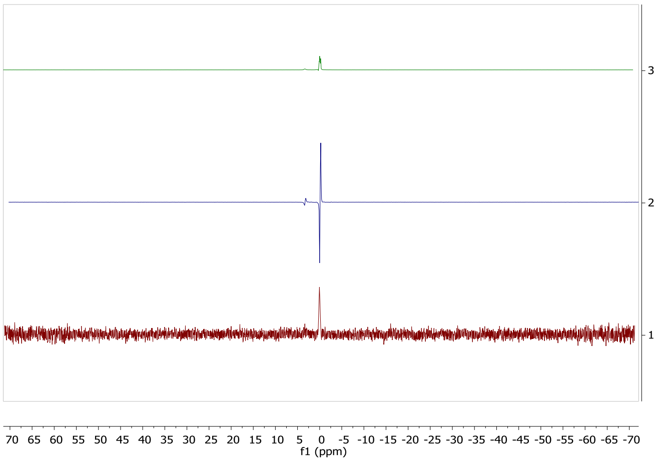

Figure S38. ^{19}F **NMR spectra of a sample consisting of** L^2 **and 1 (ratio 4:1) in** d_4**-methanol. Spectrum 1: Single scan thermally polarised spectrum (magnified 128 times relative to other spectra); 2: spectrum produced after hyperpolarisation in earth's magnetic field; 3: spectrum produced following hyperpolarisation in a µ-magnetic shield (SABRE-SHEATH)**

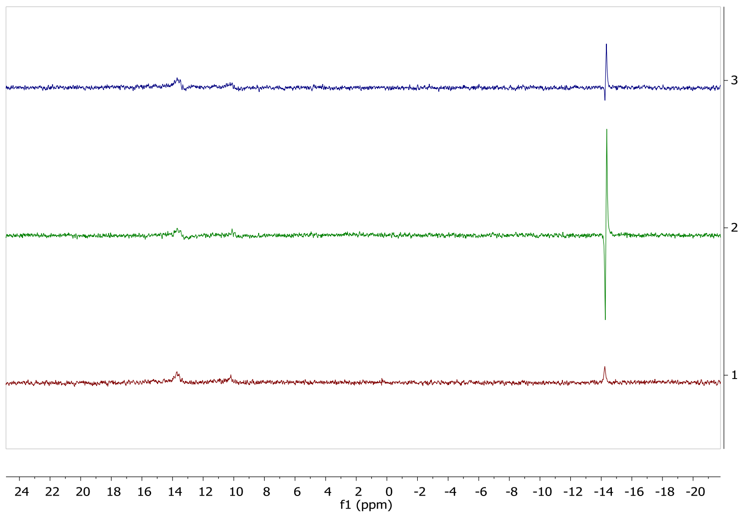

Figure S39. ^{19}**F NMR spectra of a sample consisting of L^{9} and 1 (ratio 4:1) in d$_{4}$-methanol. Spectrum 1: Single scan thermally polarised spectrum; 2: spectrum produced after hyperpolarisation in earth's magnetic field; 3: spectrum produced following hyperpolarisation in a µ-magnetic shield (SABRE-SHEATH)**

4.3 ^{19}F longitudinal relaxation times.

Longitudinal relaxation times (T_1) have been determined experimentally using the inversion recovery method for samples containing 17-fold excess of substrate to **1**. The results are presented in Table S17.

Table S17. ^{19}F longitudinal relaxation times measured at 11.7 T.

Ligand	L^2	L^4	L^6	$L^7{}_a$	$L^7{}_b$
T_1	4.46	5.51	4.91	3.16	3.28

The results show that for all compounds, the ^{19}F longitudinal relaxation times are very low, which accounts for the difference in the enhancement values obtained for ^1H and ^{19}F.

4.4 ^{19}F NMR SABRE enhancements as a function of the polarization transfer field.

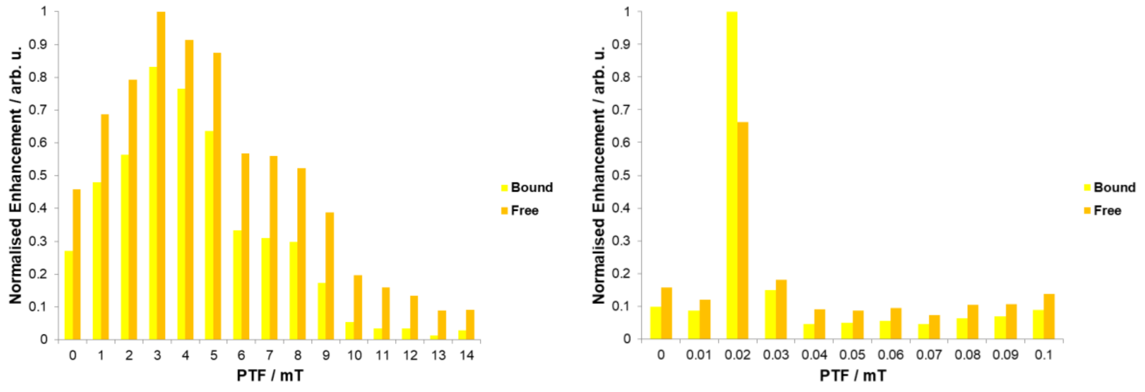

Figure S40. Normalized SABRE ^{19}F enhancements for the L^2 ^{19}F resonance as a function of PTF. Left: 0-140 mT, right: 0:0.1 mT.

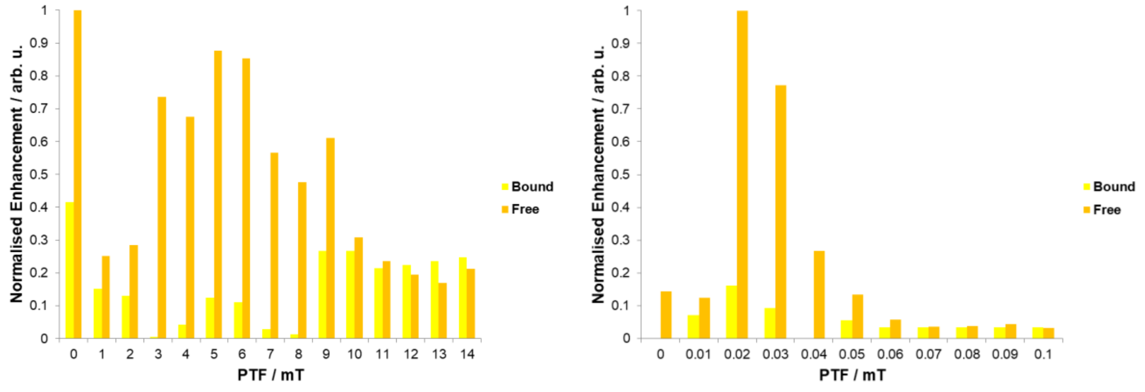

Figure S41. Normalized SABRE ^{19}F enhancements for the L^4 ^{19}F resonance as a function of PTF. Left: 0-140 mT, right: 0:0.1 mT.

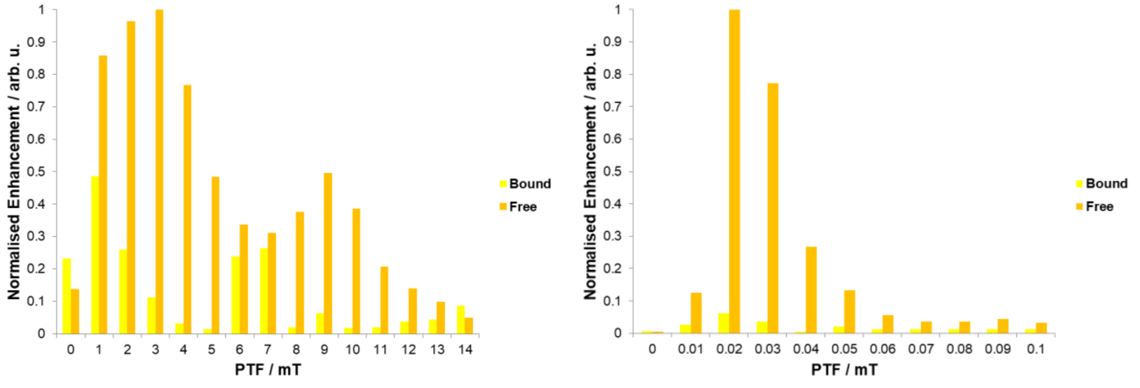

Figure S42. Normalized SABRE ^{19}F enhancements for the L^6 ^{19}F resonance as a function of PTF. Left: 0-140 mT, right: 0:0.1 mT.

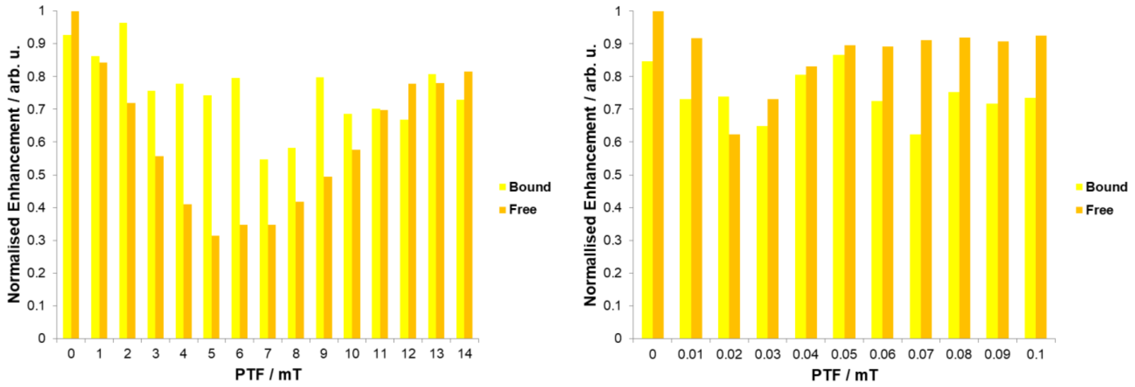

Figure S43. Normalized SABRE ^{19}F enhancements for the L^7_a ^{19}F resonance as a function of PTF. Left: 0-140 mT, right: 0:0.1 mT.

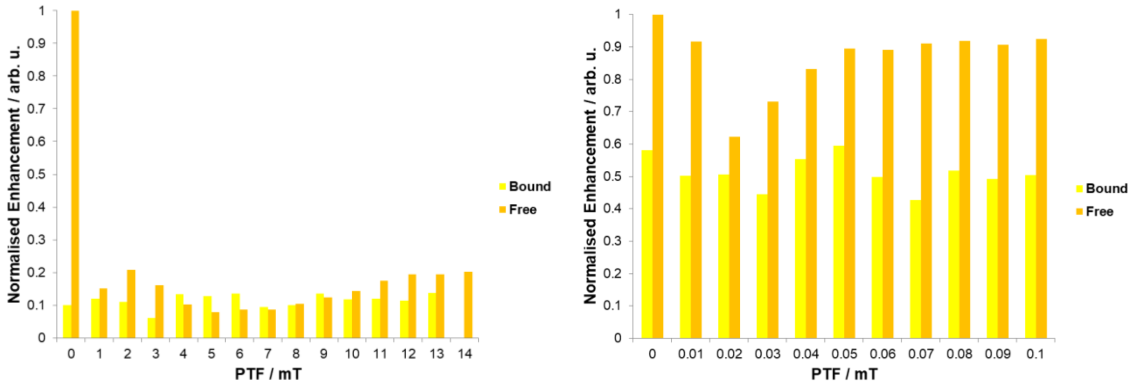

Figure S44. Normalized SABRE ^{19}F enhancements for the L^7_b ^{19}F resonance as a function of PTF. Left: 0-140 mT, right: 0:0.1 mT.

5. ^{19}F MRI Experimental Results

All the ^{19}F MRI experiments were performed on a 400 MHz (9.4 T) Bruker Advance III spectrometer (^{1}H frequency 400 MHz, ^{19}F frequency 376.5 MHz) equipped with a microimaging gradient system with a maximum amplitude of 1 T/m and a double resonance ^{13}C-^{19}F 30 mm diameter birdcage coil.

Hyperpolarized images of an axial slice located in the centre of the sample were acquired using a one shot RARE 2D protocol and the following parameters: matrix size 64x64, field of view 40 mm x 40 mm (leading to a nominal resolution of 0.625 x 0.625 mm^2/pixel) slice thickness 5 mm, echo train length 64, TE/TE_{eff}/TR: 4/4/600 ms. Images acquired in Boltzmann equilibrium conditions were recorded using the same parameters but employing a number of scans (NS) equal to 64 and a TR equal to 5 times the longitudinal relaxation time of each ligand. At least 5 experiments were performed on each sample and signal to noise (SNR) ratios were calculated for each result as the mean of the signal contained in a region of interest indicated by the user divided by the standard deviation of the noise.

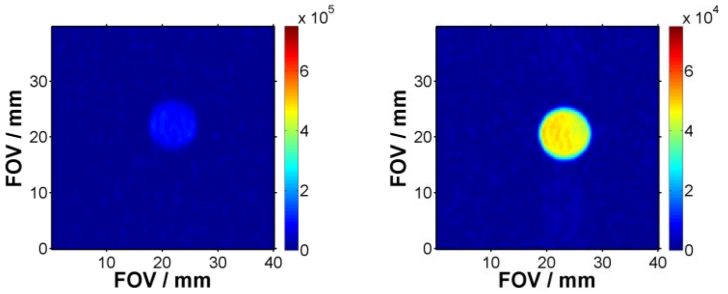

Figure S45. **^{19}F MRI 2D images of L^2. Left: Image acquired in Boltzmann equilibrium conditions, TR = 30 s, NS = 64. Right: Hyperpolarized image, TR = 600 ms, NS = 1.**

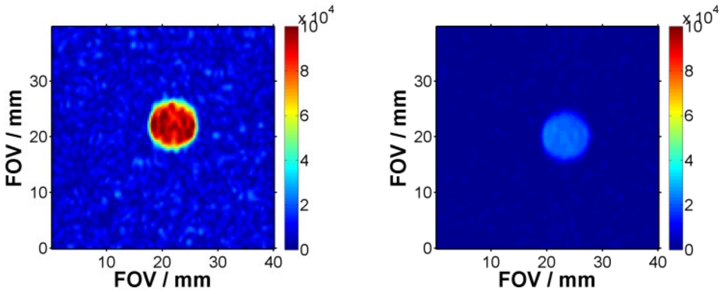

Figure S46. **^{19}F MRI 2D images of L^4. Left: Image acquired in Boltzmann equilibrium conditions, TR = 30 s, NS = 64. Right: Hyperpolarized image, TR = 600 ms, NS = 1.**

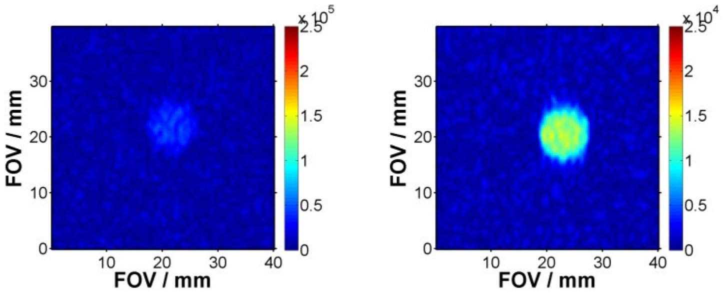

Figure S47. **^{19}F MRI 2D images of L^6. Left: Image acquired in Boltzmann equilibrium conditions, TR = 30 s, NS = 64. Right: Hyperpolarized image, TR = 600 ms, NS = 1.**

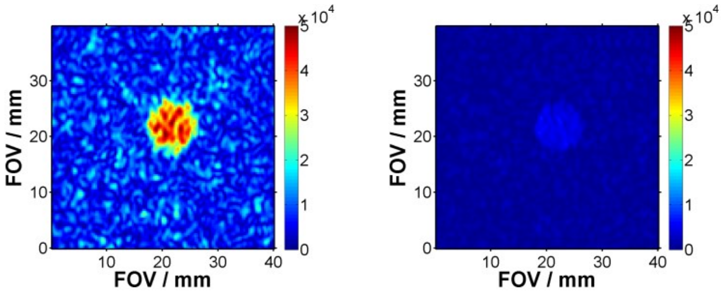

Figure S48. **^{19}F MRI 2D images of L^7_a. Left: Image acquired in Boltzmann equilibrium conditions, TR = 30 s, NS = 64. Right: Hyperpolarized image, TR = 600 ms, NS = 1.**

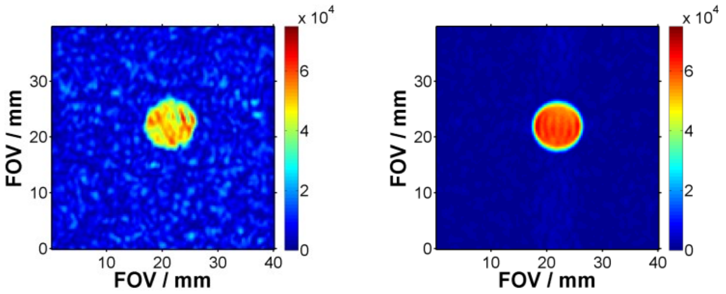

Figure S49. Images of $L^7{}_b$. Left: Image acquired in Boltzmann equilibrium conditions, TR = 30 s, NS = 64. Right: Hyperpolarized image, TR = 600 ms, NS = 1.

Table S18. Signal to noise ratios of the MRI results.

Ligand	$SNR_{thermal}$	$SNR_{thermal}$ / 1 scan	SNR_{hyp} (average)	SNR_{hyp} (maximum)	SNR gain (average)	SNR gain (maximum)
L^2	5.1	0.6	60.2	62.6	100.3	104.3
L^4	17.4	2.2	29.5	34.7	13.4	15.8
L^6	7.2	0.9	12.7	16.8	14.1	18.7
L^{7a}	1.6	0.2	5.8	6.6	2.9	3.3
L^{7b}	8.5	1.06	66.7	80.1	62.9	75.6

6. NMR Characterization

NMR characterization data for [Ir(IMes)(H)$_2$(L^2)$_3$]Cl (17-fold excess to 5 mM of 1 in MeOD) at 245 K.

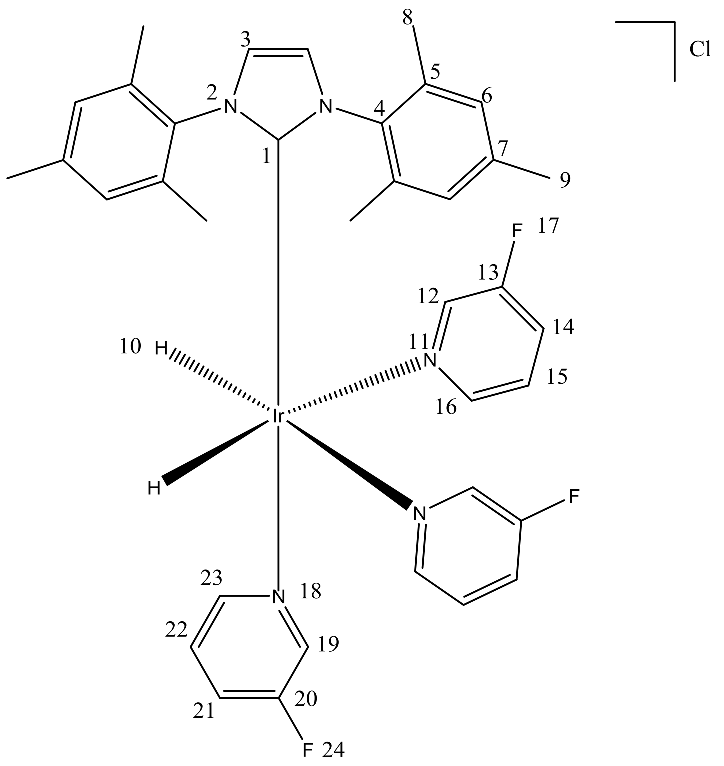

Resonance number	^{1}H (ppm)	^{13}C (ppm)	^{15}N / ^{19}F (ppm)
**1**			
**2**		-	194.69
**3**	7.23	122.71	
**4**		137.00	
**5**		135.00	
**6**	6.62	128.32	
**7**		138.45	
**8**	2.07	17.62	
**9**	2.22	19.60	
**10**	-22.85		
**11**			260.18
**12**	8.42	149.13	
**13**			
**14**	7.38	126.23	
**15**	7.72	123.74	
**16**	8.00	144.22	
**17**			-124.2
**18**			242.73
**19**	7.94	151.38	
**20**			
**21**	7.08	126.67	
**22**	7.64	123.93	
**23**	8.25	144.51	
**24**			-124.5

NMR characterization data for [Ir(IMes)(H)$_2$(L^4)$_3$]Cl and [Ir(IMes)(H)$_2$(L^4)$_2$(MeOD)]Cl (17-fold excess to 5 mM of 1 in MeOD) at 245 K.

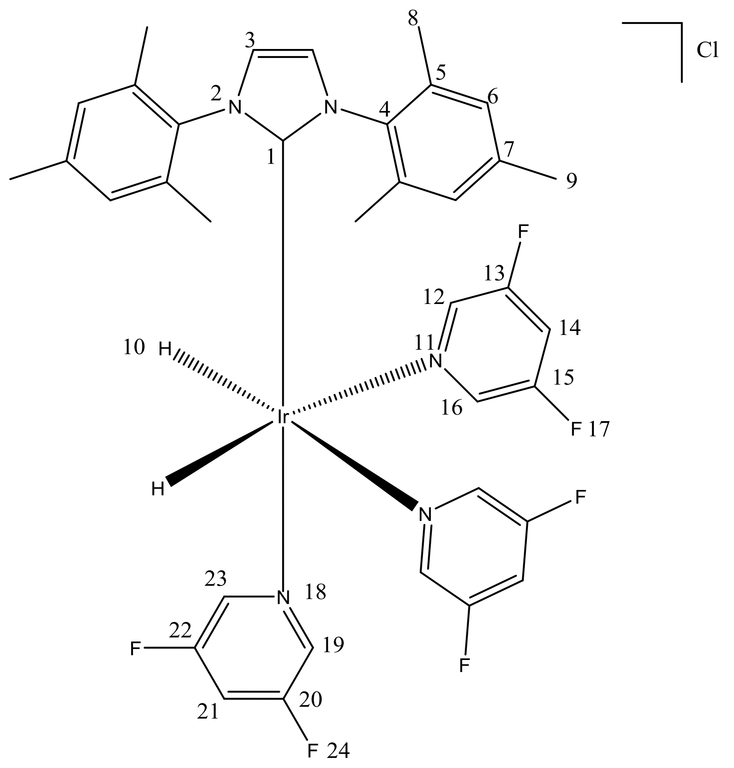

Resonance number	^{1}H (ppm)	^{13}C (ppm)	^{15}N (ppm)
**1**		148.3	
**2**			194.97
**3**	7.27	122.94	
**4**		136.8	
**5**		135.1	
**6**	6.78	128.4	
**7**			
**8**	2.09	17.59	
**9**	2.25	19.67	
**10**	-23.22		
**11**			261.6
**12,16**	8.19		
**13,15**		159.6	
**14**	7.88	112.72	
**17**			-124.35
**18**			243.8
**19,23**	8.21	141.48	
**20,22**			
**21**	7.77	112.02	
**24**			-124.77

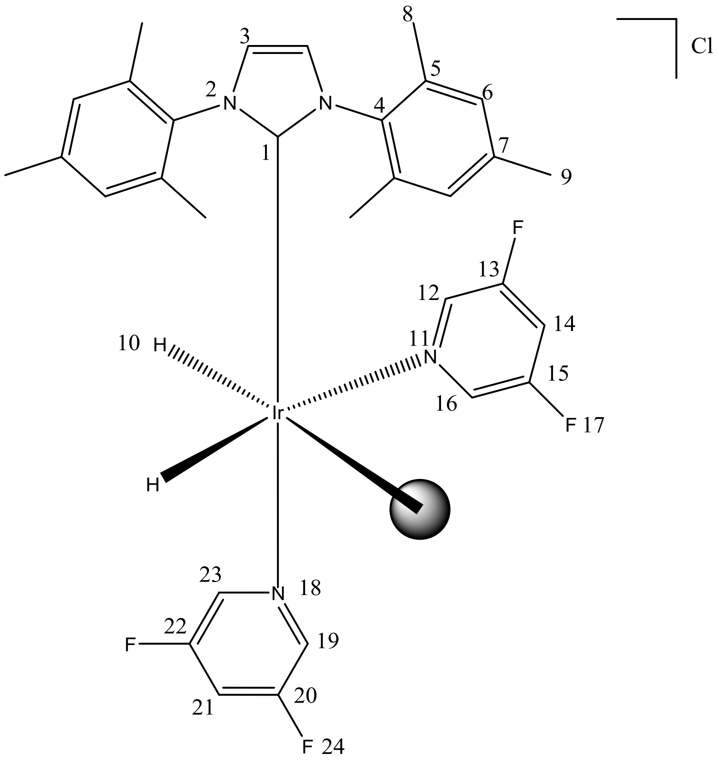

Resonance number	^{1}H (ppm)	^{13}C (ppm)	^{15}N / ^{19}F (ppm)
**1**		150.05	
**2**			193.4
**3**	7.06	121.92	
**4**		138.33	
**5**		135.5, 135.9	
**6**	6.86, 6.765	128.18, 128.25	
**7**			
**8**	2.25	17.84	
**9**	2.22, 2.20	17.68, 17.50	
**10**	-23.94, d 8.3 Hz		
**11**			269
**12,16**	8.18 br	139.65	-124.44
**13,15**		159.0, 159.3	
**14**	7.60	110.2	
**17**			-124.16
**18**			247.4
**19,23**	8.66	140.53	
**20,22**		159.0	
**21**	7.62	111.8	
**24**			-124.9

NMR characterization data for [Ir(IMes)(H)$_2$(L^6)$_3$]Cl and [Ir(IMes)(H)$_2$(L^6)$_2$(MeOD)]Cl (17-fold excess to 5 mM of 1 in MeOD) at 245 K.

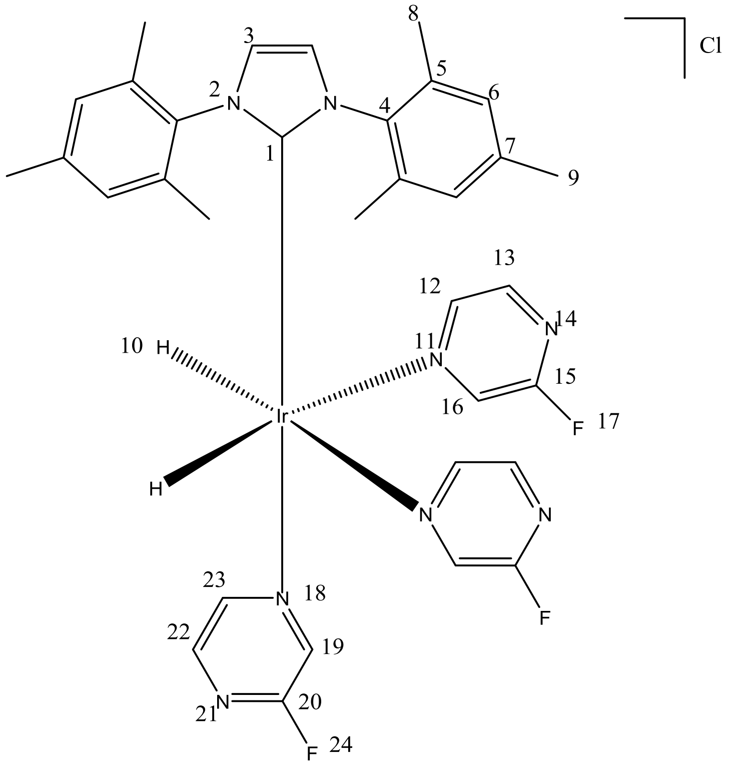

Resonance number	^{1}H (ppm)	^{13}C (ppm)	^{15}N (ppm)
**1**		148.00	
**2**			194.83
**3**	7.33	123.23	
**4**		136.53	
**5**		139.20	
**6**	6.73	128.40	
**7**		135.25	
**8**	2.09	17.60	
**9**	2.26	19.54	
**10**	-22.20		
**11**			283.70
**12**	8.52	145.54	
**13**	8.25	143.72	
**14**			290.9
**15**		161.25	
**16**	8.15	139.70	
**17**			-77.91
**18**			266.25
**19**	8.39	140.40	
**20**		147.20	
**21**			291.4
**22**	7.97	144.24	
**23**	8.10	147.28	
**24**			-78.35

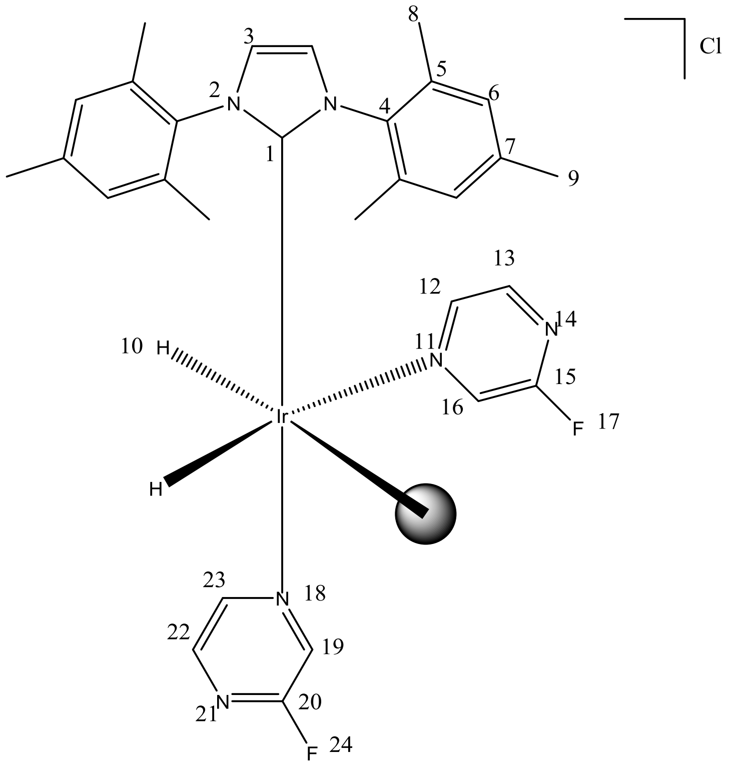

Resonance number	¹H (ppm)	¹³C (ppm)	¹⁵N (ppm)
**1**		150.72	
**2**			193.47
**3**	7.11	122.09	
**4**		136.07	
**5**		138.22, 138.43	
**6**	6.87,6.71	127.98	
**7**		135.59	
**8**	2.22, 2.18	17.60, 17.37	
**9**	2.25	19.71	
**10**	-22.73, -24.35		
**11**			272.26
**12**	7.88	142.57	
**13**	8.69	147.96	
**14**		-	287.85, 39 Hz
**15**		161.10, 233 Hz	
**16**	8.78	138.5	
**17**			-79.7
**18**			272.97
**19**			
**20**			
**21**			292.40, br
**22**			
**23**			
**24**			-80.85

NMR characterization data for [Ir(IMes)(H)$_2$(L$^7{}_a$)$_3$]Cl (17-fold excess to 5 mM catalyst precursor in MeOD) at 245 K.

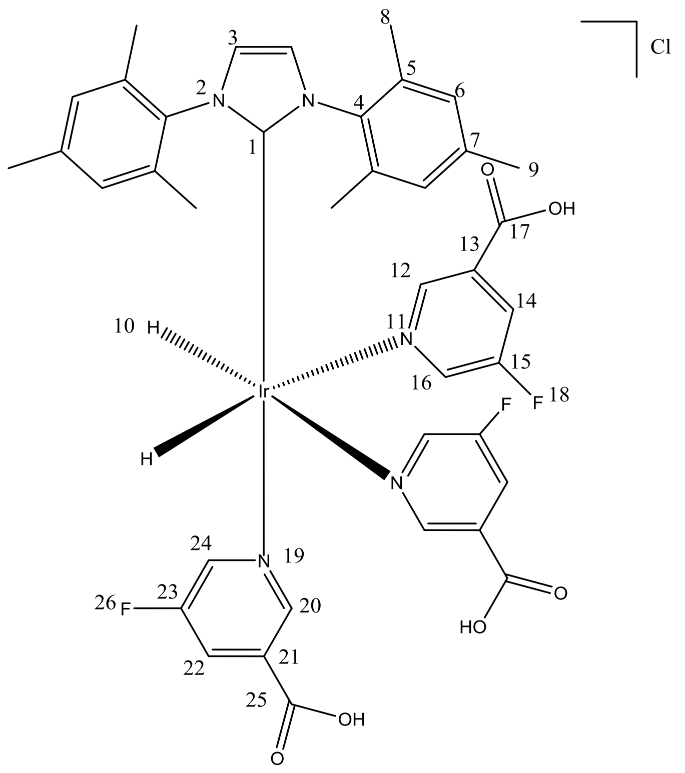

Resonance number	^{1}H (ppm)	^{13}C (ppm)	^{15}N (ppm)
**1**		149.58	
**2**			
**3**	7.29	122.97	
**4**		134.95	
**5**		136.88, 137.62	
**6**	6.73	128.53	
**7**		139.66	
**8**	2.09	17.63	
**9**	2.21	19.62	
**10**	-22.58		
**11**			259.8
**12**	8.88	151.04	
**13**		124.35	
**14**	8.28		
**15**			
**16**	8.40	146.23, d 33 Hz	
**17**			
**18**			-123.59
**19**			
**20**	8.73	153.99	
**21**			
**22**	8.15	146.2	
**23**			
**24**	8.22		
**25**			
**26**			-126.67

NMR characterization data for [Ir(IMes)(H)$_2$(L$^7{}_b$)$_3$]Cl (17-fold excess of ligand and 17-fold excess Cs_2CO_3 to 5 mM of 1 in MeOD) at 245 K.

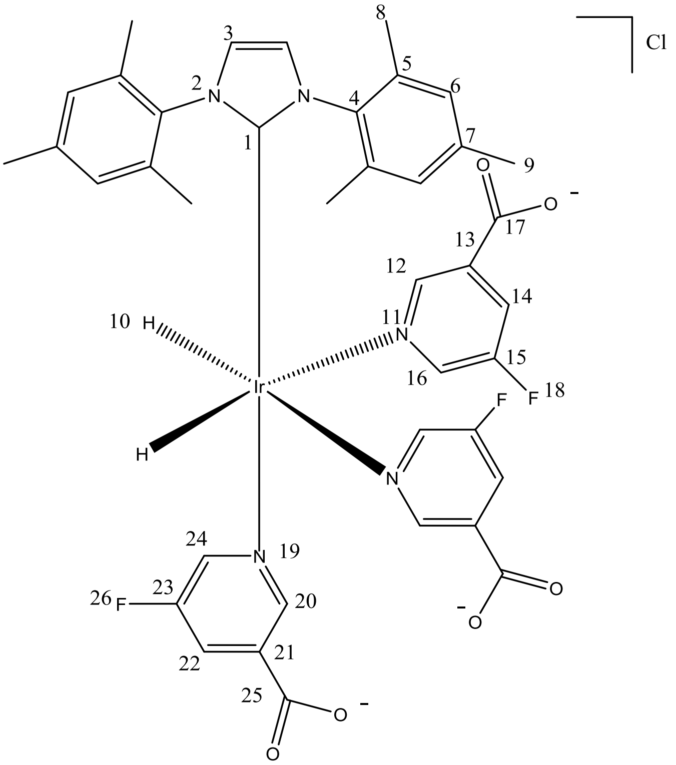

Resonance number	^{1}H (ppm)	^{13}C (ppm)	^{15}N (ppm)
**1**			
**2**			194.6
**3**	7.22	122.61	
**4**			
**5**		136.72	
**6**	6.69	128.35	
**7**			
**8**	2.08	17.76	
**9**	2.20	19.72	
**10**	-22.66	-	
**11**		-	258.0
**12**	8.87	151.00	
**13**			
**14**	7.98	123.15	
**15**			
**16**	8.15	144.3	
**17**			
**18**			-125.99
**19**			241
**20**	8.79	154.15	
**21**			
**22**	7.93		
**23**			
**24**	8.05	144.00	
**25**			
**26**			-125.67

7. References

1. O. Torres, M. Martín and E. Sola, *Organometallics*, 2009, **28**, 863-870.
2. J. Stonehouse, P. Adell, J. Keeler and A. J. Shaka, *Journal of the American Chemical Society*, 1994, **116**, 6037-6038.
3. K. Stott, J. Stonehouse, J. Keeler, T.-L. Hwang and A. J. Shaka, *Journal of the American Chemical Society*, 1995, **117**, 4199-4200.
4. N. G. Vassilev and V. S. Dimitrov, *Magnetic Resonance in Chemistry*, 2001, **39**, 607-614.
5. B. Ancian, I. Bourgeois, J.-F. Dauphin and A. A. Shaw, *Journal of Magnetic Resonance*, 1997, **125**, 348-354.
6. H. Kessler, H. Oschkinat, C. Griesinger and W. Bermel, *Journal of Magnetic Resonance (1969)*, 1986, **70**, 106-133.
7. R. E. Mewis, K. D. Atkinson, M. J. Cowley, S. B. Duckett, G. G. R. Green, R. A. Green, L. A. R. Highton, D. Kilgour, L. S. Lloyd, J. A. B. Lohman and D. C. Williamson, *Magnetic Resonance in Chemistry*, 2014, **52**, 358-369.
8. W. D. Jones, G. P. Rosini and J. A. Maguire, *Organometallics*, 1999, **18**, 1754-1760.
9. C. Runge, *Mathematische Annalen*, 1895, **46**, 167-178.
10. W. Kutta, *Z.Math.Phys.*, 1901, **46**, 435-453.
11. D. Marquardt, *Journal of the Society for Industrial and Applied Mathematics*, 1963, **11**, 431-441.
12. H. Eyring, *Chemical Reviews*, 1935, **17**, 65-77.
13. J. C. Polanyi, *Accounts of Chemical Research*, 1972, **5**, 161-168.
14. B. García, S. Ibeas and J. M. Leal, *Journal of Physical Organic Chemistry*, 1996, **9**, 593-597.
15. A. M. Olaru, M. J. Burns, G. G. R. Green and S. B. Duckett, *Chemical Science*, 2017, **8**, 2257-2266.
16. K. X. Moreno, K. Nasr, M. Milne, A. D. Sherry and W. J. Goux, *Journal of Magnetic Resonance*, 2015, **257**, 15-23.
17. M. Fekete, O. Bayfield, S. B. Duckett, S. Hart, R. E. Mewis, N. Pridmore, P. J. Rayner and A. Whitwood, *Inorganic Chemistry*, 2013, **52**, 13453-13461.
18. L. S. Lloyd, A. Asghar, M. J. Burns, A. Charlton, S. Coombes, M. J. Cowley, G. J. Dear, S. B. Duckett, G. R. Genov, G. G. R. Green, L. A. R. Highton, A. J. J. Hooper, M. Khan, I. G. Khazal, R. J. Lewis, R. E. Mewis, A. D. Roberts and A. J. Ruddlesden, *Catalysis Science & Technology*, 2014, **4**, 3544-3554.
19. G. A. Morris and R. Freeman, *Journal of the American Chemical Society*, 1979, **101**, 760-762.
